# Cell-Free Genic Extrachromosomal Circular DNA Profiles of DNase Knockouts Associated with Systemic Lupus Erythematosus and Relation with Common Fragile Sites

**DOI:** 10.3390/biomedicines12010080

**Published:** 2023-12-28

**Authors:** Daniela Gerovska, Patricia Fernández Moreno, Aitor Zabala, Marcos J. Araúzo-Bravo

**Affiliations:** 1 Computational Biology and Systems Biomedicine, Biogipuzkoa Health Research Institute, Calle Doctor Begiristain s/n, 20014 San Sebastian, Spain; daniela.gerovska@biodonostia.org (D.G.); patricia.fernandezmoreno@biodonostia.org (P.F.M.); aitor.zabalagarcia@biodonostia.org (A.Z.); 2 Basque Foundation for Science, IKERBASQUE, Calle María Díaz Harokoa 3, 48013 Bilbao, Spain; 3 Department of Cell Biology and Histology, Faculty of Medicine and Nursing, University of Basque Country (UPV/EHU), 48940 Leioa, Spain

**Keywords:** extrachromosomal circular DNA (eccDNA), systemic lupus erythematosus (SLE), Dnase1, Dnase1l3, chromosomal fragile site (CFS), genome-wide association study (GWAS), DifCir, produced per gene circle (PpGC), differentially produced per gene circle (DPpGC), plasma, liver, buffy coat

## Abstract

Cell-free extrachromosomal circular DNA (cf-eccDNA) has been proposed as a promising early biomarker for disease diagnosis, progression and drug response. Its established biomarker features are changes in the number and length distribution of cf-eccDNA. Another novel promising biomarker is a set of eccDNA excised from a panel of genes specific to a condition compared to a control. Deficiencies in two endonucleases that specifically target DNA, Dnase1 and Dnase1l3, are associated with systemic lupus erythematosus (SLE). To study the genic eccDNA profiles in the case of their deficiencies, we mapped sequenced eccDNA data from plasma, liver and buffy coat from *Dnase1* and *Dnase1l3* knockouts (KOs), and wild type controls in mouse. Next, we performed an eccDNA differential analysis between KO and control groups using our DifCir algorithm. We found a specific genic cf-eccDNA fingerprint of the Dnase1l3 group compared to the wild type controls involving 131 genes; 26% of them were associated with human chromosomal fragile sites (CFSs) and with a statistically significant enrichment of CFS-associated genes. We found six genes in common with the genic cf-eccDNA profile of SLE patients with DNASE1L3 deficiency, namely *Rorb*, *Mvb12b*, *Osbpl10*, *Fto*, *Tnik* and *Arhgap10*; all of them were specific and present in all human plasma samples, and none of them were associated with CFSs. A not so distinctive genic cf-eccDNA difference involving only seven genes was observed in the case of the Dnase1 group compared to the wild type. In tissue—liver and buffy coat—we did not detect the same genic eccDNA difference observed in the plasma samples. These results point to a specific role of a set of genic eccDNA in plasma from *DNase* KOs, as well as a relation with CFS genes, confirming the promise of the genic cf-eccDNA in studying diseases and the need for further research on the relationship between eccDNA and CFSs.

## 1. Introduction

DNA degradation is critical for healthy organism development and survival. The regular development and healthy function of humans and mice requires two families of endonucleases, Dnase1 and Dnase2. Deoxyribonuclease 1, Dnase1, and deoxyribonuclease 1 like 3, Dnase1l3, belong to the Dnase1 family, with Dnase1 expressed primarily in the digestive tract and in blood, where it helps clean free DNA from the circulation. Dnase1l3 is expressed by dendritic cells and macrophages in the liver and spleen, in myeloid cells in blood, and in neurons, with an unknown role in the latter [1]. A disease associated with deficiencies of Dnase1 and Dnase1l3 in mouse is systemic lupus erythematosus (SLE). SLE is characterized by antibody generation, activation and complement by these antibodies, inflammation, and organ damage, with the secreted DNases, Dnase1 and Dnase1l3, providing the greatest protection from autoantibody formation [1].

Cell-free (cf) extrachromosomal circular DNA (eccDNA) has a potential clinical application as a biomarker in cancer [2] and other diseases such as diabetes [3], inflammatory bowel disease [4], and amyotrophic lateral sclerosis [5], among others. EccDNA features such as changes in the number of the eccDNAs, changes in the length frequency distributions of the eccDNA or differential abundance of eccDNA from certain genes [3,6,7] have been considered as biomarkers. Previous work studying the relationship of nucleases and eccDNA characteristics in plasma using knockout (KO) mouse models with a deficiency in Dnase1 or Dnase1l3 found that cf-eccDNA *Dnase1l3*^−/−^ exhibited larger size distributions than in wild type mice [8]. Anyway, current studies still suffer from a limited number of patients and samples to validate the statistical power of such features to discriminate between conditions.

Common fragile sites (CFSs) are specific genomic loci that are difficult to replicate and correspond to regions of genomic instability. Upon replication stress, CFSs form gaps or breakages, a phenomenon called CFS expression, which correlate positively with chromosomal rearrangement and copy number variation, and where under-replicated DNA subsequently undergoes mitotic DNA synthesis, MiDAS [9]. Based on their frequency, CFSs encompass two classes: the rare CFSs, which are present in the 5% of the population, and common CFSs present in nearly all the population. Intrinsic characteristics of the CFSs are the overlap with very large and active genes, with many of these large genes involved in neurological development [10,11]. The CFSs are known to be conserved features of human and mouse chromosomes [11]. CFSs have been extensively studied due to their important pathophysiological relevance in diseases such as cancer and neurological disorders, for reviews of CFSs see [12,13]. The possible relationship between CFSs and eccDNA, and CFSs and SLE has not been studied so far.

Here we explored the possibility that the DNase KO groups differ from the wild type (WT) group in their genic eccDNA profiles in mouse plasma and tissue samples, as in the case of cf-eccDNA from SLE patients with *DNASE1L3* mutations [14]. We performed a circular differential analysis based on the split read signal of the eccDNA using our DifCir algorithm [15] of circulomics data from WT, *Dnase1l3*^−/−^ (from here on denoted as Pla-KO1l3) and *Dnase1*^−/−^ (here denoted as Pla-KO1) eccDNA purified plasma samples, comparing the KOs with the WT (here denoted as Pla-WT), control samples. We compared the obtained top-ranked genes producing differential eccDNA in plasma with the eccDNA produced by the same genes in KO and healthy control tissue—buffy coat and liver—samples. We aimed to look for commonalities between the genic eccDNA profiles of the two SLE mouse models and humans monogenic for SLE with DNASE1L3 deficiency. Furthermore, we explored the relationship between genic eccDNA production and the excision of the eccDNA from CFSs in both human SLE and mouse models.

## 2. Methods

### 2.1. Samples and Data

Plasma DNA samples from 12 WT, 11 *Dnase1*^−/−^, and 11 *Dnase1l3*^−/−^ mice [8] were used to study the genic cell-free (cf) eccDNA profiles of the two DNase KOs compared to the WT. For the plasma samples, we used short-read sequenced data of eccDNA libraries obtained with a tagmentation (Tn)-based preparation protocol [16].

Additionally, we used 5 WT, 5 *Dnase1*^−/−^ and 5 *Dnase1l3*^−/−^ liver, and 6 WT, 4 *Dnase1*^−/−^, and 5 *Dnase1l3*^−/−^ buffy coat samples to assess the eccDNA excising behavior of the genes by characterizing the genic cf-eccDNA profiles of the KOs in liver and buffy coat tissues. For the tissue samples, we used short-read sequenced data of eccDNA libraries obtained using both a Tn- and a rolling circle amplification (RCA)-based preparation protocol [16].

All data have been downloaded from the European Genome-Phenome Archive (EGA) hosted by the European Bioinformatics Institute (EBI) (accession EGAS00001005873) [8].

### 2.2. Mapping of eccDNA and Quantification of Produced per Gene Circles/eccDNAs (PpGCs)

Mapping and quantification of produced per gene eccDNAs (PpGCs) was performed as described previously [15]. EccDNA reads were mapped with Circle_Finder [17,18] using as parameters the mm10 build of the mouse genome and a minNonOverlap threshold between two split reads of 10 bp. Clusters of eccDNAs within a distance smaller than *D_min_* = 10 bp were coalesced and the number of the split reads detecting them were summed as split reads of the merged eccDNAs. EccDNAs with less than two split reads were excluded. EccDNA annotations were accomplished with bedtools intersect using Genecode gene coordinates. EccDNA sizes were assessed from mitochondria-free DNAs, setting 10 Kbp as the cutoff for all small size circles. Next, the number of split reads of all those eccDNAs that carried the same gene or fragment of a gene were added to obtain the unscaled produced per gene eccDNA (PpGC*_i_*) sum for each gene *i*. For scaling by gene length, each PpGC*_i_* was multiplied by a scale factor *L_Max_*/*L_i_*, where *L_Max_* is the length of the longest gene found in the dataset, and *L_i_* is the length of the gene *i*. Finally, data equalization was performed using the log_2_(PpGC + 1) transformation of the scaled PpGCs to obtain the final PpGCs.

### 2.3. Identification of Differentially Produced per Gene eccDNAs (DPpGCs)

We used our method—DifCir—for the differential analysis of sequenced purified eccDNA data based on their split read signal [15]. We calculated the mean values of the PpGCs for each group of replicates and selected as differentially produced per gene eccDNAs (DPpGCs) those whose absolute difference of the means between the two groups was more than a selection threshold *θ*_DPpGC_ = one-fold change (FC) on the log_2_ scale. Finally, we selected the statistically significant DPpGCs based on Student’s t-test using a significance threshold of α_DPpGC_ = 0.05.

### 2.4. Calculation of the Chromosome Enrichment of DPpGCs

The chromosome enrichment of the DPpGCs is calculated as the percentage of the difference of the ratio of DPpGCs in each chromosome minus the ratio of DPpGCs in the whole genome as follows: Δ*c* = 100(*Nd_c_*/*N_c_* − *Nd_g_*/*N_g_*), where *Nd_c_* is the number of DPpGCs of chromosome *c*, *N_c_* is the number of eccDNAs of the chromosome *c*, *Nd_g_* is the number DPpGCs in the whole genome, and *N_g_* is the number of eccDNAs in the whole genome. The Δ*c* value shows the percentage of enrichment or depletion (for negative cases) of DPpGCs of each chromosome with respect to the percentage of DPpGCs in the whole genome. Since a high percentage of difference Δ*c* does not ensure statistical significance, to assess the significance of the results, the hypergeometric test was used with mid-*p*-values. The significantly enriched chromosomes are labeled with stars ∗, where ∗ means that 0.01 ≥ mid- *p*-value > 0.001, ∗∗ means that 0.001 ≥ mid-*p*-value > 0.0001, etc. A higher number of asterisks corresponds to higher statistical significance of the results.

### 2.5. Finding Common Genes between up-DPpGCs in Plasma from Dnase1l3^−/−^ Mice and SLE Patients with DNASE1L3 Deficiency

We intersected the list of genes identified as statistically significant up-DPpGCs in the mouse *Dnase1l3*^−/−^ group compared to WT here with the list of 267 statistically significant (significance level α_DPpGC_ = 0.01) in the SLE patients with DNASE1L3 deficiency (Pla-SLE) compared to a healthy control group (Pla-HC) from [14].

### 2.6. Finding Common DPpGCs with Chromosomal Fragile Site (CFS) Genes

The CFS genes were taken from the database of fragile sites in human chromosomes (HumCFS) [19], downloaded on 6 November 2023. The CFSs were stratified in 6 categories based on the drug used to destabilize the DNA (5-azacytidine, aphidicolin, BrdU, distamycinA, folic acid, and the pool of the five drugs). For each of the CFSs, we collected the genes located in each CFS locus and annotated them with the corresponding drug associated with the CFS. To calculate the statistical enrichment of the CFS genes, we used the sixth group constituted by pooling all of the CFS genes affected by any of the CFS drugs. To assign the original human genes names to mouse genes names, we used the NCBI HomoloGene database. The statistical significance of the enrichment of the CFSs was interrogated using the hypergeometric test using a significance threshold of α_CFS_ = 0.05.

### 2.7. Finding Common DPpGCs with GWAS for ‘Systemic Lupus Erythematosus’

The Genome-Wide Association Study (GWAS) data were downloaded on 5 February 2023 from the GWAS Catalog of the National Human Genome Research Institute (NHGRI) and European Bioinformatics Institute (EBI). The data in the GWAS Catalog used are currently mapped to human genome assembly GRCh38.p13 and dbSNP Build 154. The statistical significance of the enrichment of any of the diseases in the GWAS Catalog was interrogated using the hypergeometric test with a significance threshold of α_GWAS_ = 0.05.

## 3. Results

### 3.1. Workflow

**Figure 1:**
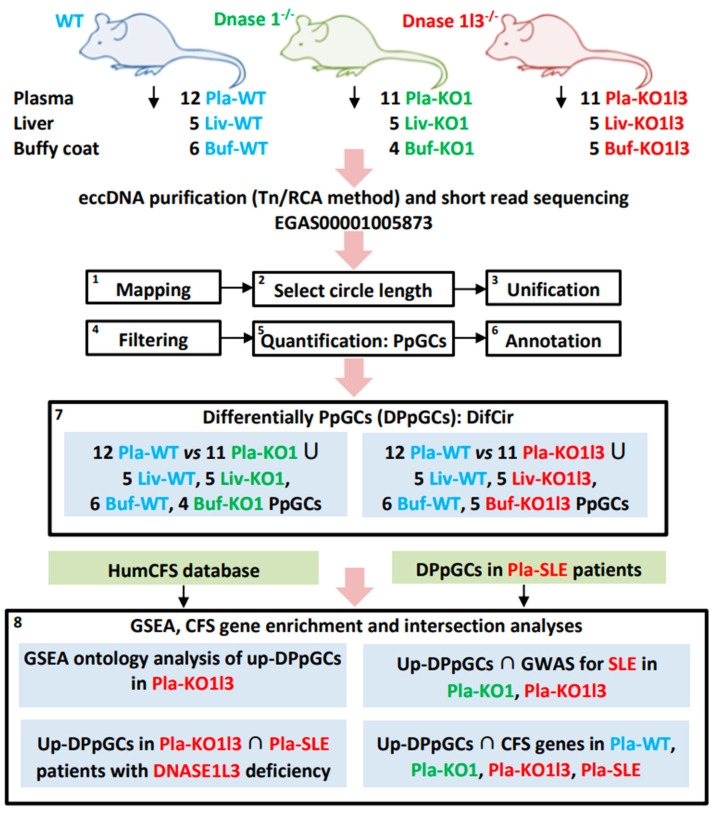
Experimental setup and computational analysis workflow of the cell-free and tissue circulomics data from *Dnase1*^−/−^, *Dnase1l3*^−/−^, and wild type (WT) mice from Sin et al. [8]. The computational analysis includes mapping, annotation, and quantification—using PpGCs—of eccDNAs, in plasma, liver, and buffy coat, followed by the calculation of DPpGCs for Pla-KO1 compared to Pla-WT and for Pla-KO1l3 compared to Pla-WT. The statistically significant up-DPpGCs in the two directions in the two comparisons were cross-checked with the PpGCs in liver and buffy coat obtained through the Tn and RCA methods, and annotated for genes from CFSs and according to the agent used to induce the CFSs, followed by calculation for CFS gene enrichment. Additionally, a functional enrichment analysis of the up-DPpGCs in Pla-KO1l3 vs. Pla-WT was performed with GSEA. A comparison between the up-DPpGCs in Pla-KO1l3 and in SLE patients with DNASE1L3 deficiency was implemented. Blue, green and red colors mark WT, Pla-KO1 and Pla-KO1l1 (and patients with Pla-SLE with DNASE1L3 deficiency), respectively.

We aimed to obtain and analyze the genic cf-eccDNA profiles of *Dnase1* and *Dnase1l3* KOs compared to wild type mice (WT). While Sin et al. [8] asked the research question what was the effect of the nucleases on the eccDNA properties like their abundance and size distribution, the question about possible differences in the way they affect the excision of eccDNA from different genes remained. Here, we processed circulomics data of cell-free (cf)-eccDNA from Pla-WT, *n* = 12, Pla-KO1 (*Dnase1*^−/−^), *n* = 11, and Pla-KO1l3 (*Dnase1l3*^−/−^), *n* = 11, groups from Sin et al. [8]. EccDNA has been isolated, purified and short-read sequenced from plasma, liver and buffy coat of WT mice, mice with *Dnase1* knockout (KO1), and mice with *Dnase1l3* knockout (KO1l3). EccDNA libraries from plasma samples were constructed using a tagmentation (Tn)-based method, while for the tissue samples of liver and buffy coat, both rolling circle amplification (RCA) and Tn have been used [8]. This was followed by mapping, annotation, and quantification of eccDNAs, and quantification of produced per gene circles (PpGCs) for the plasma, liver and buffy coat samples. Next, the calculation of differentially PpGCs (DPpGCs) was performed for Pla-KO1 compared to Pla-WT and for Pla-KO1l3 compared to Pla-WT. The statistically significant up-DPpGCs in the two directions in the two comparisons were cross-checked with the PpGCs in liver and buffy coat obtained through the Tn and RCA methods. Functional enrichment analysis of the up-DPpGCs in Pla-KO1l3 vs. Pla-WT was performed with the gene sets from GSEA. To study the extent to which the mouse model reflects the human SLE disease, a comparison of the up-DPpGCs in Pla-KO1l3 vs. Pla-WT and the up-DPpGCs in SLE patients with DNASE1L3 deficiency vs. healthy controls has been implemented. Additionally, the intersection with the term ‘systemic lupus erythematosus’ from the GWAS database was performed to annotate in our results the genes that are already associated with SLE. The up-DPpGCs in the four comparisons were checked for genes from chromosomal fragile sites (CFSs) and annotated as such, and additionally according to the agent used to induce the CFSs. The data and schematic workflow of this work are presented in detail in Figure 1.

### 3.2. The Number of cf-eccDNAs Does Not Discriminate between the DNase KOs and WT

**Figure 2:**
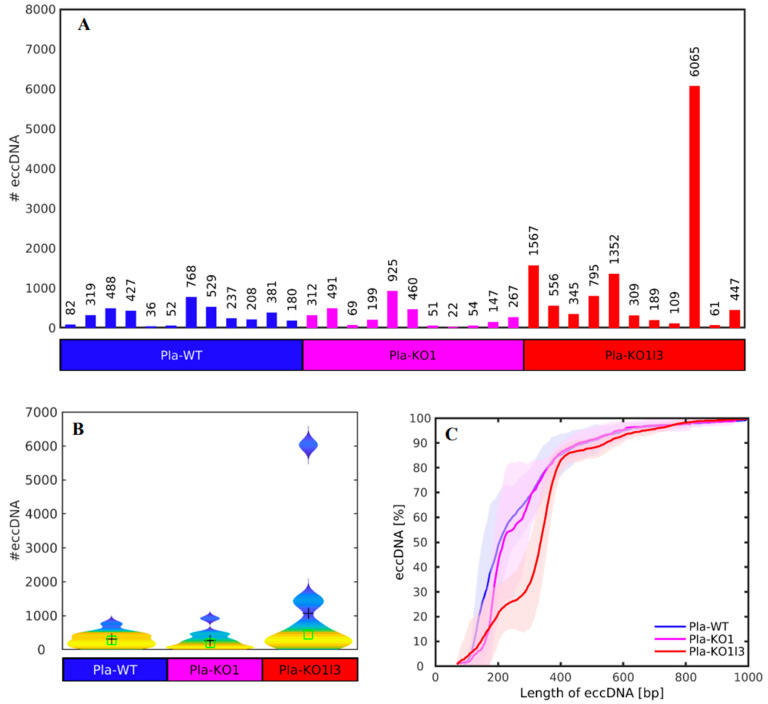
Number (#) and length distribution of the unique eccDNAs mapped in the three mouse plasma sample groups. (**A**) Number of unique eccDNAs. (**B**) Violin plots of the number of eccDNAs. The black crosses and the green squares mark the position of the mean and the median of the distributions, respectively. (**C**) Cumulative frequency distribution of the lengths of the eccDNAs in the Pla-WT (wild type). The shadowed blue, magenta, and red regions cover the standard deviation of the enrichment across the Pla-WT, Pla-KO1 and Pla-KO1l3 groups, respectively. Pla-WT, *n* = 12, Pla-KO1 (*Dnase1*^−/−^), *n* = 11, and Pla-KO1l3 (*Dnase1l3*^−/−^), *n* = 11, groups.

First, we studied whether the number or the sizes of the cf-eccDNAs could be used to discriminate between the two DNase KOs and the WT. The number and the length distribution of the cf-eccDNAs mapped by us are presented in Figure 2. We observed no significant differences between the number (Figure 2A) and size distribution (Figure 2B) of eccDNAs in Pla-WT and Pla-KO1 samples. The Wilcoxon rank sum test confirmed that the 1.1338-fold difference is not statistically significant between Pla-KO1 (mean 272.5 ± sem 81.6), where sem is the standard error of the mean, and Pla-WT (mean 308.9 ± sem 63.6) at 1% significance level with a *p*-value 0.5254.

In the case of Pla-KO1l3, there is a higher variability in the number of eccDNAs than in the other conditions, with a sample with an extremely high number of 6065 eccDNAs (Figure 2A); however, the difference in relation to Pla-WT is not statistically significant, and the sample with a high number of eccDNAs could be considered as an outlier based on the violin plots of the distributions of the numbers of eccDNAs (Figure 2B). The Wilcoxon rank sum test did not indicate that the 0.2881-fold difference between Pla-KO1l3 (1072.3 ± 520.7) and Pla-WT is statistically significantly different at a 1% significance level with a *p*-value 0.1507. Neither indicate that the 0.2541-fold difference in the number of eccDNAs in Pla-KO1l3 and Pla-KO1 is statistically significant at a 1% significance level with a *p*-value 0.0759.

In the case of the other classical eccDNA marker, the length of the eccDNAs, the percentages of eccDNAs with lengths ranging from 0 to 1000 bases is very similar for Pla-WT and Pla-KO1 (Figure 2C). In the case of Pla-KO1l3, there is a slight decrease in plasma KO1l3 eccDNAs with lengths ranging from 200 to 400 bases. However, the percentages of lengths become similar for the three analyzed cases for eccDNAs with lengths greater than 400 bases. Altogether, Figure 2, as well as a previous detailed analysis of these features [8], show that the length and frequency do not discriminate confidently between the groups.

### 3.3. Existing but Scarce Genic cf-eccDNA Differences between Dnase1 KO and WT

**Figure 3:**
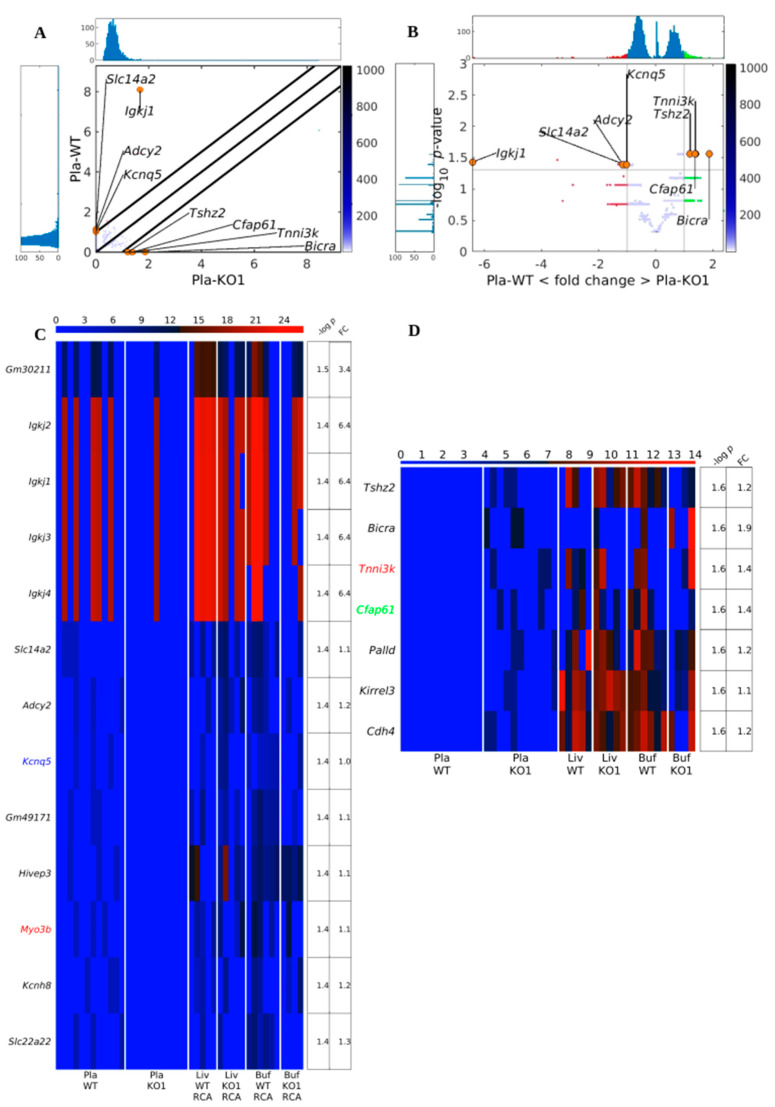
Scatter plot (**A**) and volcano plot (**B**) of the PpGCs in Pla-KO1 vs. Pla-WT samples. In (**B**), the vertical black lines are the boundaries of the log_2_(FC) 1 thresholds for the eccDNA production levels between the groups. The horizontal black line is the 1.3-boundary of the *p*-value in –log_10_ scale (corresponding to *p*-value = 0.05 in linear scale). PpGCs beyond these thresholds were inferred to be significantly distinct from control eccDNAs and denominated as DPpGCs. Their distributions are visualized with histograms. Red and green dots mark PpGCs with abundance below and above the FC threshold. Orange dots indicate selected DPpGCs. Heatmaps of the (**C**) 13 genes that led to up-DPpGCs in WT relative to Pla-KO1 and (**D**) 7 genes that led to up-DPpGCs in Pla-KO1 relative to WT in decreasing order of significance. The PpGCs in the liver samples, Liv-WT-RCA and Liv-KO1-RCA, and in the buffy coat samples, Buf-WT-RCA and Buf-KO1-RCA, are given in the heatmaps as a comparison for eccDNA production in the corresponding tissues. The color bars codify the value of PpGCs on the log_2_ scale. A higher value corresponds to a redder color. The –log_10_(*p*-value) and the absolute value of the log_2_ of the fold change (FC) in the DPpGCs are presented in a table to the right of the heatmaps. The gene names at the left side of the heatmaps are colored in red, green, blue, cyan, and magenta for genes associated with CFSs induced by aphidicolin, folic acid, BrdU, 5-azacytidine, and distamycinA, respectively.

The traditional approaches, numbers and lengths of eccDNAs, to discriminate between sample groups using circulomics data did not find differences between *Dnase1* KO and WT in plasma samples. Such techniques are not very sensitive since they pool all the potential information of the eccDNAs in a global number for each sample; hence, we developed our DifCir method based on gene-centric information, produced per gene circles (PpGCs). Therefore, to assess whether some genes in the mouse genome were particularly prone to form eccDNA in the *Dnase1* KO group compared to the WT group, we calculated the PpGCs for the Pla-KO1 and Pla-WT samples. The paired scatter plot in Figure 3A shows certain differences in PpGCs in the Pla-KO1 vs. Pla-WT groups, and the volcano plot in Figure 3B shows that some of these differences are statistically significant. For a log_2_ fold change ≥1 and significance level threshold α_DPpGC_ = 0.05, we identified 13 up-DPpGCs in Pla-WT compared to Pla-KO1 (*Gm30211*, *Igkj1-4*, *Slc14a2*, *Adcy2*, *Kcnq5*, *Gm49171*, *Hivep3*, *Myo3b*, *Kcnh8* and *Slc22a22*), as depicted in the heatmap of Figure 3C, and 7 up-DPpGCs in Pla-KO1 related to Pla-WT (*Tshz2*, *Bicra*, *Tnni3k*, *Cfap61*, *Palld*, *Kirrel3*, and *Cdh4*), as depicted in the heatmap of Figure 3D. Among the latter, *Tshz2*, teashirt zinc finger homeobox 2, is a tumor suppressor that inhibits metastasis [20], and *Bicra*, BRD4 interacting chromatin remodeling complex associated protein, enables transcription regulator activator activity. To assess the relation between the DPpGCs and chromosomal fragile sites (CFSs), we marked in the heatmaps the gene names associated with CFSs confirmed by different drugs. We found two CFS-associated genes *Myo3b* (aphidicolin-induced CFS) and *Kcnq5* (BrdU-induced CFS) in Pla-WT and another two, *Tnni3k* (aphidicolin-induced CFS) and *Cfap61* (folic acid-induced CFS) in Pla-KO1, i.e., 15% and 29% of the up-DPpGCs, respectively. However, for the comparisons in the two directions, the number of DPpGCs in CFSs is not statistically significantly enriched (Table 1). Furthermore, to track and compare the production of eccDNA in tissue with available circulomics data for the up-DPpGCs in Pla-WT and Pla-KO1, we represented in the heatmaps the PpGCs in liver and buffy coat tissue from the WT and KO1 samples for the libraries obtained with the RCA method (Figure 3C,D). We observed that the up-DPpGCs in the Pla-WT vs. Pla-KO1 and Pla-KO1 vs. Pla-WT groups do not correlate with the PpGCs in liver and buffy coat, where the PpGCs for the genes up-producing cf-eccDNA in the two comparisons have generally high values in both WT and KO liver and buffy coat samples. We chose to show in the heatmaps in Figure 3C,D the PpGCs in tissue samples from libraries obtained with RCA method since using the RCA method for tissues led to the mapping of a high number of eccDNAs, while the Tn method resulted in mapping of very few eccDNAs. The same heatmaps with PpGCs from liver and buffy coat obtained with the Tn method are represented in Supplementary Figure S1A,B.

### 3.4. Distinctive and Specific Genic cf-eccDNA Profile of Dnase1l3 KO Compared to WT

**Figure 4:**
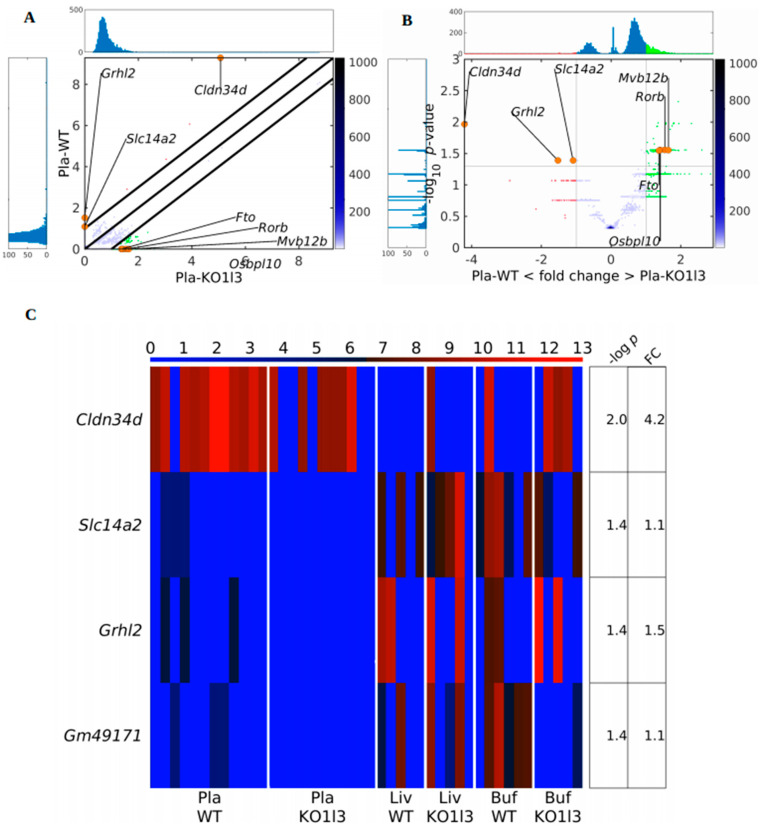
Scatter plot (**A**) and volcano plot (**B**) of the PpGCs in Pla-KO1l3 vs. Pla-WT samples. In (**A**), the parallel black lines are the boundaries of the log_2_(FC) 1 thresholds for the eccDNA production levels between the groups. In (**B**), the vertical black lines are the boundaries of the log_2_(FC) 1 thresholds for the eccDNA production levels between the groups. The horizontal black line is the 1.3-boundary of the *p*-value on the –log_10_ scale (corresponding to *p*-value = 0.05 in linear scale). PpGCs beyond these thresholds were inferred to be significantly distinct from control eccDNAs and denominated as DPpGCs. Their distributions are visualized in histograms. Red and green dots mark PpGCs with abundance below and above the FC threshold. Orange dots indicate selected DPpGCs. (**C**) Heatmap of the four genes that led to up-DPpGCs in Pla-WT relative to Pla-KO1l3 in decreasing order of significance. The PpGCs in the liver samples, Liv-WT-RCA and Liv-KO1l3-RCA, and in the buffy coat samples, Buf-WT-RCA and Buf-KO1l3-RCA, are given in the heatmaps as a comparison for the eccDNA production in the corresponding tissues. The color bars codify the value of PpGCs on the log_2_ scale. A higher value corresponds to a redder color. The –log_10_(*p*-value) and the absolute value of the log_2_ of the fold change (FC) of the DPpGCs are presented in a table to the right of the heatmap. The gene names to the left of the heatmaps are colored in red, green, blue, cyan, and magenta for genes associated with CFSs induced by aphidicolin, folic acid, BrdU, 5-azacytidine, and distamycinA, respectively.

**Figure 5:**
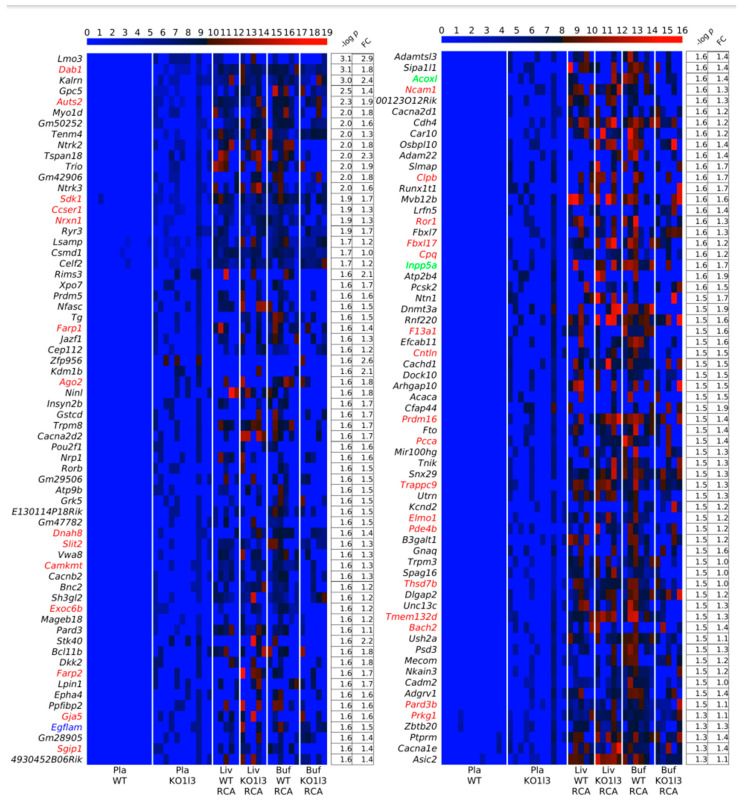
Heatmaps of the 131 genes that led to up-DPpGCs in Pla-KO1l3 compared to WT in decreasing order of significance. The PpGCs in the liver samples, Liv-WT-RCA and Liv-KO1-RCA, and in the buffy coat samples, Buf-WT-RCA and Buf-KO1-RCA, are given in the heatmaps as a comparison for the eccDNA production in the corresponding tissues. The color bars codify the value of PpGCs on the log_2_ scale. A higher value corresponds to a redder color. The –log_10_(*p*-value) and the absolute value of the log_2_ of the fold change (FC) of the DPpGCs are presented in a table to the right of the heatmaps. The gene names to the left of the heatmaps are colored in red, green, blue, cyan, and magenta for genes associated with chromosomal fragile sites (CFSs) induced by aphidicolin, folic acid, BrdU, 5-azacytidine, and distamycinA, respectively.

In the plasma *Dnase1l3* KO vs. WT case, the traditional approaches, comparisons of numbers and lengths of eccDNAs, as in the case of *Dnase1* KO vs. WT, did not find significant differences between the sample groups using circulomics data. Additionally, in the comparison of Pla-KO1 vs. Pla-WT, we found only scarce difference with our gene-centric approach. However, to assess whether any of the genes in the mouse genome were particularly prone to form cf-eccDNA in the *Dnase1l3* KO group compared to the WT group, we calculated the PpGCs for the Pla-KO1l3 and Pla-WT samples. The paired scatter plot in Figure 4A shows differences in PpGCs in the Pla-KO1l3 and Pla-WT samples. For a log_2_ fold change ≥1 (FC ≥ 2 on the linear scale) and a −log_10_(*p*-value) ≥ 1.3, i.e., a significance level threshold α_DPpGC_ = 0.05, we identified 131 up-DPpGCs in Pla-KO1l3 related to Pla-WT (Figure 4B and Figure 5) and 4 up-DPpGCs in Pla-WT compared to Pla-KO1l3 (Figure 4B,C), namely, *Cldn34d*, *Slc14a2*, *Grhl2* and *Gm49171*. The statistically most significant up-DPpGC in Pla-KO1l3 is *Lmo3*, LIM domain only 3, which is predicted to enable ion binding activity, with a *p*-value of 0.00079 (Figure 5), while the top up-DPpGC in Pla-WT compared to Pla-KO1l3 is *Cldn34d*, claudin 34D, which is involved in bicellular tight junction assembly and cell adhesion, with a much higher *p*-value of 0.01080 (Figure 4C). Anyway, *Cldn34d* shows high PpGC values even in Pla-KO1l3 samples and thus is not specific for Pla-WT. Next, we checked whether the genic circulomics difference in plasma correlates with that in liver and buffy coat tissues. We chose to show in the heatmaps in Figure 4C and Figure 5 the PpGCs in tissue samples from libraries obtained with the RCA method since using this method in tissues led to the mapping of a high number of eccDNAs, while the Tn method resulted in mapping very few eccDNA. The same heatmaps of PpGCs in liver and buffy coat obtained with the Tn method are represented in Supplementary Figures S2 and S3. Interestingly, for the genes up-producing cf-eccDNA in Pla-KO1l3 and Pla-WT, the PpGCs do not differentiate between the KO and the WT groups in both liver and buffy coat tissue. In conclusion, the genic cf-eccDNA profile of Pla-KO1l3 vs. Pla-WT is distinctive with a high number of genes; it is specific, with cf-eccDNA from these genes produced generally only in Pla-KO1l3 and none in Pla-WT; and it does not correlate with a difference from the same genes in liver and buffy coat tissues.

### 3.5. Association of up-DPpGCs in Pla-KO1l3 with CFSs

We checked our list of up-DPpGCs in Pla-KO1l3 for genes from chromosomal fragile sites (CFSs), characterized as common and rare, as genomically unstable regions, which are hot-spots for deletions and other alterations. We found that disabled-1, *Dab1*, the second ranked one, has been characterized as a large CFS gene that is mapped within the FRA1B CFS and inactivated in multiple cancers [10,21], while *AUTS2* (rank 5) maps in FRA7J, *NRXN1* (rank 16) in FRA2D, *PRKG1* (127) in FRA10C [10], and *SDK1* (14) in FRA7B [22]. We checked the other up-DPpGCs in the HumCFS database [19], as of 6 October 2023, and found an additional 29 up-DPpGCs as genes associated with CFSs. Thus, 34 of the 131 up-DPpGCs in Pla-KO1l3, or 26%, are genes mapped to characterized CFSs. This percentage is similar to the 29% of up-DPpGCs in Pla-KO1. Anyway, while in Pla-KO1 the up-DPpGCs are not enriched in CFS genes, in PlaKO1l3, this enrichment is statistically significant with a *p*-value of 8.1695·10^−9^ (Table 1). The CFS-associated genes are listed in Table 2 together with their rank as up-DPpGCs and their association with a CFS in humans and the corresponding mouse chromosome, and additionally marked in Figure 5 with a color corresponding to the agent with which they have been discovered. Of the six genes on human chromosome 1, five map to mouse chromosome 4 and one to mouse chromosome 3. The three genes on human chromosome 8 map to mouse chromosome 15. The rest of the human CFC genes have a more disperse mapping to mouse chromosomes.

Next, we calculated the statistical enrichment of the CFS genes. We found that the genic cf-eccDNA profile of *Dnase1l3*^−/−^ mice compared to WT mice is statistically significantly enriched in CFS genes (Table 1).

### 3.6. Association of up-DPpGCs in Pla-KO1l3 with SLE in the GWAS catalog

We made an intersection of the up-DPpGCs in Pla-KO1l3 with the genes for ‘systemic lupus erythematosus’ in the GWAS catalog and found three genes in common, namely, *JAZF1*, *DOCK10*, and *BACH2*. Gateva et al. [23] found the promoter region of *JAZF1*, juxtaposed with another zinc finger gene 1, as a confirmed SLE locus (rs849142). We found *JAZF1* to be the only gene common with the GWAS term ‘systemic lupus erythematosus and systemic sclerosis’. *DOCK10*, dedicator of cytokinesis 10, belongs to a cytokinesis protein family, whose members are guanosine nucleotide exchange factors for Rho GTPases. It has been confirmed as a SLE locus (rs11462616) in an Egyptian population [24]. Sheng et al. [25] suggested that overexpression of the transcriptional regulator *BACH2* represses Th9 cell differentiation by suppressing *IRF4* expression in SLE patients, and that it might be a potential target for SLE treatment. *BACH2*, BTB and CNC homology 1, basic leucine zipper transcription factor 2, is the only one of the three genes associated with a common CFS, FRA6G.

### 3.7. Chromosomal Landscaping of up-DPpGCs in Pla-KO1l3 Compared to Pla-WT

**Figure 6:**
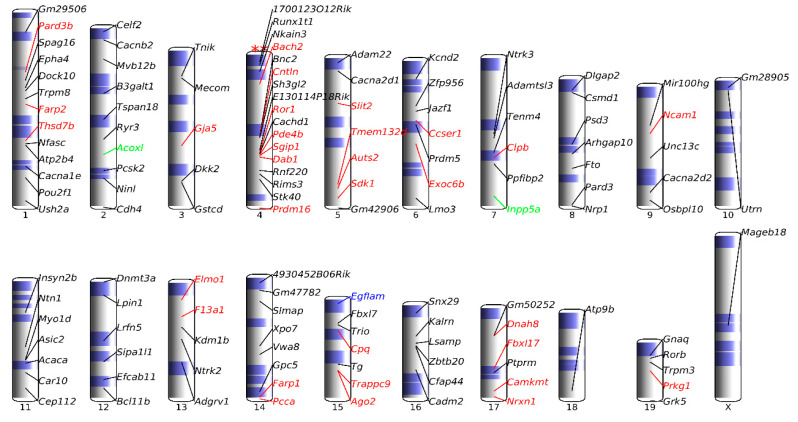
Chromosomal landscape of genomic loci giving rise to statistically significant up-DPpGCs in Pla-KO1l3 compared to Pla-WT. The colored genes are associated with chromosomal fragile sites (CFSs): red, green, blue, cyan, and magenta are for genes associated with CFSs induced by aphidicolin, folic acid, BrdU, 5-azacytidine, and distamycinA, respectively. Chromosome Y has not been included since there were no DPpGCs excised from this chromosome. ∗∗ over chromosome 4 means that it has a significant enrichment of up-DPpGCs with 0.001 ≥ mid-*p*-value > 0.0001.

Our DifCir method revealed a high number of up-DPpGCs in *Dnase1l3* KO vs. WT plasma. To obtain a better insight in the distribution of the DPpGCs and the DPpGCs that are CFS genes across the chromosomes, we performed chromosomal landscaping of the distribution of these up-DPpGCs, which showed that chromosome 4 is statistically significantly enriched in DPpGCs in *Dnase1l3* KO mice (Figure 6). Additionally, we observed in the chromosomal landscape that all the CFS-related genes on chromosome 4 have been produced by aphidicolin. On chromosome 4, there seems to be a cluster of four CFS-associated genes (*Ror1*, *Pde4b*, *Sgip1*, and *Dab1*). The first three are from the human FRA1L CFS, and the last one is from the FRA1B CFS (Table 2). *Cachd1* belongs to the same cluster and is not a known CFS gene; anyway, its location between three FRA1L genes suggests that it might also be a FRA1L gene, and at the same time, *Dab1* does not exactly belong to the cluster. On chromosome 14, *Farp* and *Pcca* belong to FRA13D, and on chromosome 15, *Trappc9* and *Ago2* associate with FRA8D (Table 2).

### 3.8. Visualization of up-DPpGCs in Pla-KO1l3, eccDNA Excising Loci and Coverage in All Plasma Samples

**Figure 7:**
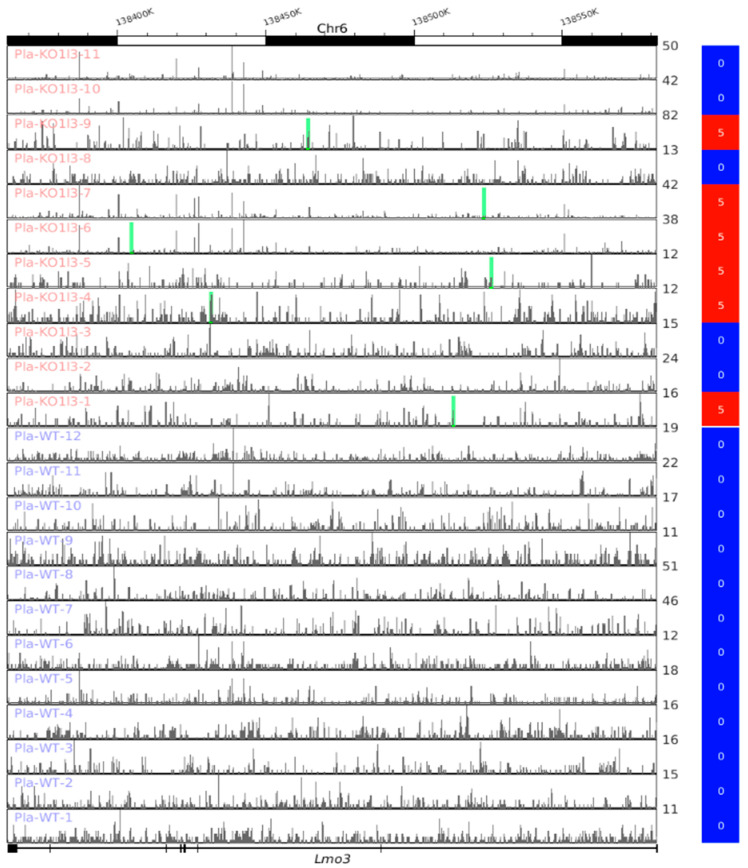
Track plots of the loci the top-ranked up-DPpGC in Pla-KO1l3 compared to WT, *Lmo3*, and the corresponding gene coverage in all the plasma samples from the two groups. Each horizontal line represents the length of a gene. The green bars represent the excision loci of the eccDNA. The color bars to the right codify the value of PpGCs on the log_2_ scale.

We explored the relation between the eccDNAs leading to the up-DPpGCs in Pla-KO1l3 and the coverage of the associated genes through depicting simultaneously the genomic coordinates of the gene from which the eccDNAs were excised, the coverage in that gene in all samples, and the value of the quantity of PpGCs for the gene and the sample. Figure 7 shows the top up-DPpGCs in Pla-KO1l3, *Lmo3*, illustrative in that the eccDNA forming the Pla-KO1l3 genic profile carries fragments of genes rather than whole genes, that these fragments do not have the same coordinates, and that most of the coverage, if not all, is not related to mapped eccDNA. In the case of *Lmo3*, only one unique eccDNA has been excised per sample in the Pla-KO1l3 samples where eccDNA has been mapped, i.e., samples 1, 4–7, and 9. The track plots of the next 10 up-DPpGCs in the ranking are shown in Supplementary Figures S5–S14. An example of more than one eccDNA sequence excised from a gene is *Auts2* (Supplementary Figure S7), where Pla-KO1l3 samples 1, 2, 4, 6 and 9 have three, one, one, one and eight eccDNAs, respectively, but the PpGC values corresponding to these samples are five, four, three, three and six, due to the different numbers of split reads mapping each unique eccDNA sequence.

### 3.9. Gene Set Enrichment Analysis of up-DPpGCs in Pla-KO1l3

**Figure 8:**
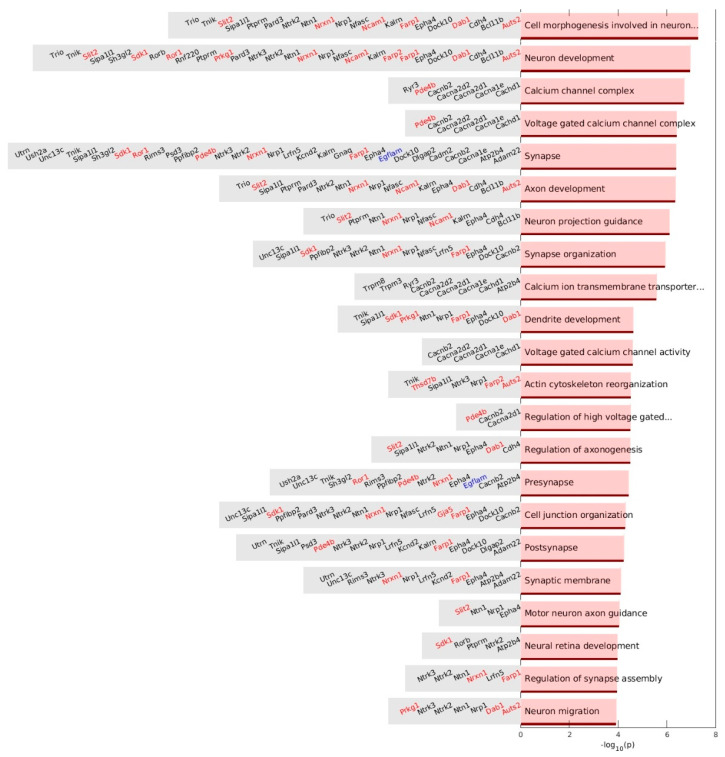
GSEA terms from the enrichment analysis of up-DPpGCs in Pla-KO1l3. (**Right**) Bar plots of the –log_10_(*p*-value) of the significantly enriched terms. (**Left**) List of genes in the significant GSEA enrichment terms. The colored genes are associated with chromosomal fragile sites (CFSs): red, green, blue, cyan, and magenta are for genes associated with CFSs induced by aphidicolin, folic acid, BrdU, 5-azacytidine, and distamycinA, respectively.

We performed a gene ontology (GO) enrichment analysis using the gene set enrichment analysis (GSEA) sets for systematic functional associations of the genes related to the up-DPpGCs. The top enriched ontology term of the up-DPpGCs in Pla-KO1l3 is ‘cell morphogenesis involved in neuron differentiation’ due to the detection of the up-DPpGCs in 20 genes: *Auts2*, *Bcl11b*, *Cdh4*, *Dab1*, *Dock10*, *Epha4*, *Farp1*, *Kalrn*, *Ncam1*, *Nfasc*, *Nrp1*, *Nrxn1*, *Ntn1*, *Ntrk2*, *Pard3*, *Ptprm*, *Sipa1l1*, *Slit2*, *Tnik*, and *Trio* (Figure 8). We checked whether some terms are enriched in CFS genes. The genes associated with neural-related GSEA terms have a high percentage of CFSs: ‘cell morphogenesis involved in neuron differentiation’ has 30% CFS genes and ‘neuron development’ has 36% CFS genes. Only two terms, ‘calcium ion transmembrane transporter activity’ and ‘voltage-gated calcium channel activity’, are represented by sets of genes that have no CFS-associated genes (Figure 8).

Additionally, we performed a GO enrichment analysis separately for Molecular functions, Biological processes and Cellular components, as presented in Supplementary Figures S14–S16. The top term for Molecular functions is ‘calcium ion transmembrane transporter activity’, which does not have any CFS-associated gene. Interestingly, among the GO Molecular function terms are ‘Rho guanyl-nucleotide exchange factor activity’ (Supplementary Figure S14) with the genes *Dock10*, *Farp1*, *Farp2*, *Kalrn*, and *Trio*. An understanding of how guanine nucleotide exchange factors (GEFs) and GTPase-activating proteins (GAPs) are regulated and interact with their GTPase targets is providing new opportunities for therapeutic development in immune-mediated inflammatory diseases [26]. The top term for Biological processes is ‘regulation of axonogenesis’, and among the terms is ‘regulation of GTPase activity’ with the genes *Epha4*, *Farp1*, *Farp2*, *Gnaq*, *Kalrn*, *Ntrk2*, *Ntrk3*, *Prkg1*, *Sipa1l1*, and *Slit2* (Supplementary Figure S15). A KALRN variant and risk allele rs1444766-G are associated with SLE. The *PRKG1* rs7897633 variant has been previously identified as the top hit in patients of European ancestry with SLE and high IFN-α levels compared to those with low circulating IFN activity [27]. Slit2 glycoprotein has been found to be a potential biomarker for renal impairment in SLE patients [28].

The first statistically significantly enriched term for GO Cellular components is ‘synapse’ with gene members *Adgrv1*, *Asic2*, *Cadm2*, *Cep112*, *Dlgap2*, *Egflam Epha4*, *Farp1*, *Kcnd2*, *Nrxn1*, *Ntrk2*, *Pde4b*, *Psd3*, *Rims3*, *Sdk1*, *Sipa1l1*, *Unc13c*, *Ush2a*, *and Utrn*, while the second statistically significantly enriched term is ‘voltage-gated calcium channel complex’ with members *Cacna1e*, *Cacna2d1*, *Cacna2d2*, *Cacnb2*, and *Pde4b* (Supplementary Figure S16). SLE T cells exhibit activation signaling anomalies, including a defective Ca^(2+)^ response and increased nuclear factor of activated T-cells, NFAT, nuclear translocation. Nicolaou et al. [29] reported a defect in Kv1.3 trafficking to the immunological synapse (IS) of SLE T cells that might contribute to the Ca^(2+)^ defect and proposed that Kv1.3 trafficking abnormalities contribute to the altered distribution of Ca^(2+)^ signaling in SLE T cells, with these defects explaining in part the T cell hyperactivity and dysfunction documented in SLE patients.

### 3.10. Common up-DPpGCs in Pla-KO1l3 and Pla-SLE Patients

Next, we aimed to compare the gene content of the genic cf-eccDNA profile from the Dnase1l3^−/−^ SLE mouse model and the genic cf-eccDNA profile of SLE patients with DNASE1L3 deficiency we identified in a previous work [14]. Common up-DPpGCs in Pla-KO1l3 with up-DPpGCs identified in human SLE plasma samples are *Rorb*, *Mvb12b*, *Osbpl10*, *Fto*, *Tnik*, and *Arhgap10*. Nandakumar et al. [30] identified the multivesicular body protein MVB12b as a target for TANK-binding kinase 1 phosphorylation, which is essential for the sorting of DNA into extracellular vesicles (EVs) and stimulation of bystander cells to stimulate the cGAS-STING pathway. Thus, MVB12b was identified as a messenger responsible for packing and exporting DNA fragments in exosomes, and it was found that turning down the messenger, rather than spreading an immune signal, sends an alarm signal to the tissue needing protection, thus providing perspectives in relation to the treatment of autoimmune diseases such as SLE. TNIK/MAP4K7 (Traf2- and Nck-interacting kinase) functions as an activator of the Wnt signaling pathway, and its inhibition has been reported to have antitumor activity [31].

### 3.11. Associations of up-DPpGCs in Pla-SLE with CFSs

**Figure 9:**
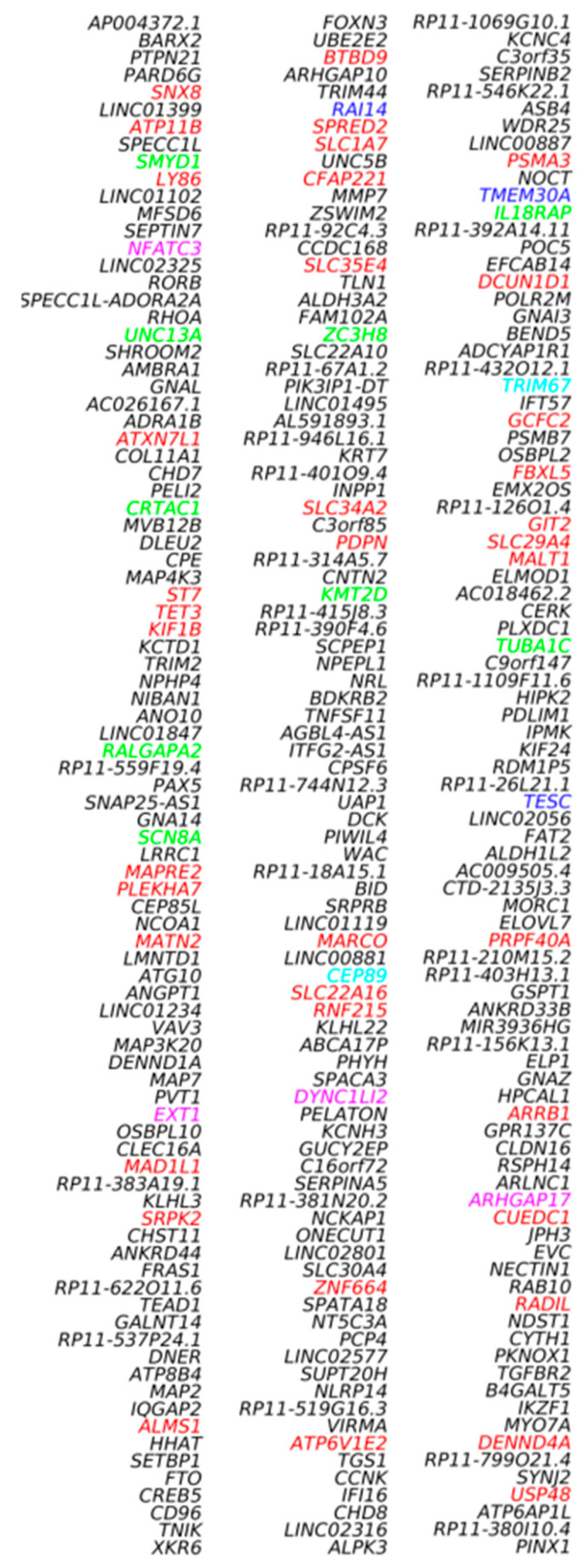
List of the up-DPpGCs in cf-eccDNA from SLE patients with DNASE1L3 deficiency compared to healthy controls in decreasing order of statistical significance. The colored genes are associated with chromosomal fragile sites (CFSs) according to the HumCFS database [19]. The color of the gene names corresponds to the chemical agent used to induce the CFS: cyan: 5-azacytidine; red: aphidicolin; blue: BrdU; magenta: distamycinA; and green: folic acid [19].

We checked the up-DPpGCs in SLE patients with DNASE1L3 deficiency for CFS genes and found that 56 out of the 267 genes, i.e., approximately 21%, are CFS genes (Figure 9 and Table 1). These CFS genes have been denoted with the color of the chemical agent used for their validation. Additionally, we checked for the statistically significant enrichment of the CFS-affected genes and found that the up-DPpGCs are indeed enriched in CFS genes (Table 1).

## 4. Discussion and Conclusions

Dnase1l3 deficiency leads to autoimmunity. Systemic lupus erythematosus (SLE) with a complete Dnase1l3 deficiency causing pediatric onset in humans is distinct from SLE in general in that it shows no gender bias, has increased incidence of lupus nephritis and anti-neutrophil cytoplasmic bodies. Though associations between Dnase1 polymorphisms and SLE have been made in humans, causality in human systems has not been proved, with Dnase1 showing clinical improvement in some SLE patients but notably not in lupus nephritis patients [1]. Here, we found a distinctive genic eccDNA profile of *Dnase1l3*^−/−^ mice compared to WT mice, with some eccDNAs being from the same genes as in the pediatric SLE patients with DNASE1L3 deficiency, namely *Rorb*, *Mvb12b*, *Osbpl10*, *Fto*, *Tnik*, and *Arhgap10*. Additionally, we identified an existent but not so discriminating genic eccDNA profile of *Dnase1*^−/−^ mice compared to WT. The difference in the genic eccDNA profiles might be related to their different associated SLE types.

In the case of the Pla-KO1 comparison, we found more up-DPpGCs in the wild type group, Pla-WT, than in the Pla-KO1 group. Contrarily, in the Pla-KO1l3 study, we found more up-DPpGCs in the Pla-KO1l3 group compared to the Pla-WT group. Interestingly, for both Pla-KO1 and Pla-KO1l3, we found higher percentages of CFSs in the KOs than in the WT, which in the Pla-KO1l3 case were statistically significant, pointing to a relationship between the genes excising eccDNA in the KOs and CFSs; a relationship that is not observed in the WT.

Extrachromosomal circular DNA in cancer (ecDNAs) frequently carry oncogenes and multi-drug resistance genes. A multitude of research suggests that a decrease in the number of ecDNAs as well as the amplified genes might result in a reversal of the cancer phenotype. For example, Yu et al. [32] provided a direct relationship between the loss of double minutes (DMs) and a decrease in ovarian cancer malignancy, showing that the drug gemcitabine decreases the number of DMs. Xu et al. [3] investigated the characteristics and dynamics of circulating eccDNAs in type 2 diabetes mellitus (T2DM) patients undergoing short-term intensive insulin therapy (SIIT) for inducing long-term glycemic remission. Genic cf-eccDNA profiles before and after treatment offer the possibility to follow the effect of drugs not only at the level of number of eccDNAs but also at the more precise level of actual genes and CFSs that are affected by the drug. Common CFSs are frequently affected in cancer and can be found in nearly all healthy individuals, opening possible avenues in the relation between CFSs and ecDNAs.

In conclusion, here, for the first time, we found commonalities between the genic cell-free circulomics profiles of a mouse model of SLE, *Dnase1l3*^−/−^, and of the human disease itself, monogenic SLE with DNASE1L3 deficiency. The common features are: firstly, both exhibit a genic cf-eccDNA profile with a high number of genes that is specific for the disease group; secondly, the two genic cf-eccDNA profiles share six common genes; and thirdly, both mouse model and human SLE profiles contain similar percentages of genes from CFSs. In comparison, the SLE mouse model, *Dnase1*^−/−^, shows a genic cf-eccDNA profile with very few genes.

## Figures and Tables

**Table 1:** Enrichment of CFSs in DPpGCs between DNase KOs and controls in mouse plasma and human samples. The percentages are the ratios of the numbers of CFSs in genes multiplied by 100 and divided by the number of DPpGCs. The *p*-value of the statistical significance is calculated by the mid *p*-values of the hypergeometric distribution. The statistically significant cases are marked in boldface. WT wild type, HC healthy control.

Species	DNase Mutation	Control	Up-DPpGC	Percentage	*p*-Value
Mouse	KO1	WT	WT	15.38%	0.2189
Mouse	KO1	WT	KO1	28.57%	0.0730
Mouse	KO1l3	WT	WT	0%	0.6578
**Mouse**	**KO1l3**	**WT**	**KO1l3**	**25.95%**	**8.1695·10^−9^**
**Human**	**DNASE1L3**	**HC**	**KO1l3**	**20.97%**	**5.298·10^−11^**

**Table 2:** Human homologs of the up-DPpGCs in Pla-KO1l3 compared to Pla-WT, their rank as up-DPpGCs, their belonging to a certain chromosomal fragile site (CFS) in humans and their corresponding mouse chromosome. In boldface are marked the cases with more stable human-to-mouse chromosome mapping.

Gene	Rank of DPpGC	CFS	Mouse Chromosome
***DAB1***	**2**	**FRA1B**	**4**
*AUTS2*	5	FRA7J	5
*SDK1*	14	FRA7B	5
*CCSER1*	15	FRA4E	6
*NRXN1*	16	FRA2D	17
*FARP1*	26	FRA13D	14
***AGO***	**31**	**FRA8D**	**15**
*DNAH*	45	FRA6H	17
*SLIT2*	46	FRA4D	5
*CAMKMT*	48	FRA2S	17
*EXOC6B*	52	FRA2E	6
*FARP2*	58	FRA2J	1
*GJA5*	62	FRA1F	3
*EGFLAM*	63	FRA5A	15
***SGIP1***	**65**	**FRA1L**	**4**
*ACOXL*	69	FRA2B	2
*NCAM1*	70	FRA11G	9
*CLPB*	78	FRA11H	7
***ROR1***	**82**	**FRA1L**	**4**
*FBXL17*	84	FRA5F	17
***CPQ***	**85**	**FRA8B**	**15**
*INPP5A*	86	FRA10A	7
*F13A1*	92	FRA6B	13
*CNTLN*	94	FRA9G	4
***PRDM16***	**100**	**FRA1A**	**4**
*PCCA*	102	FRA13D	14
***TRAPPC9***	**106**	**FRA8D**	**15**
*ELMO1*	109	FRA7C	13
***PDE4B***	**110**	**FRA1L**	**4**
*THSD7B*	115	FRA2F	1
*TMEM132D*	118	FRA12E	5
*BACH2*	119	FRA6G	4
*PARD3B*	126	FRA21	1
*PRKG1*	127	FRA10C	19

## Data Availability

This study makes use of data generated by The Chinese University of Hong Kong (CUHK) Circulating Nucleic Acids Research Group, as reported by Sin et al. in JCI insight (doi: 10.1172/jci.insight.156070). The data have been deposited in the European Genome-Phenome Archive hosted by the European Bioinformatics Institute (accession EGAS00001005873.).

## Supplementary Materials

The following supporting information can be downloaded at: https://www.mdpi.com/article/10.3390/biomedicines12010080/s1. Supplementary Materials file.
