# Supplementary material for: Cell-Free Genic Extrachromosomal Circular DNA Profiles of DNase Knockouts Associated with Systemic Lupus Erythematosus and Relation with Common Fragile Sites
Source: Biomedicines. 2023 Dec 28;12(1):80. doi: 10.3390/biomedicines12010080 (PMC10813657; doi:10.3390/biomedicines12010080)
Supplement: Supplementary file 1 [file biomedicines-12-00080-s001.zip › biomedicines-2698632-supplementary.pdf]

SUPPLEMENTARY MATERIAL

Cell-Free Genic Extrachromosomal Circular DNA Profiles of DNase Knockouts Associated with Systemic Lupus Erythematosus and Relation with Common Fragile Sites

Daniela Gerovska [1], Patricia Fernández Moreno [1], Aitor Zabala [1] and Marcos J. Araúzo-Bravo [1,2,3*]

[1] Computational Biology and Systems Biomedicine, Biogipuzkoa Health Research Institute, Calle Doctor Begiristain s/n, 20014 San Sebastian, Spain; daniela.gerovska@biodonostia.org (D.G.); patricia.fernandezmoreno@biodonostia.org (P.F.M.); aitor.zabalagarcia@biodonostia.org (A.Z.); mararabra@yahoo.co.uk;marcos.arauzo@biodonostia.org (M.J.A.-B.)
[2] Basque Foundation for Science, IKERBASQUE, Calle María Díaz Harokoa 3, 48013 Bilbao, Spain
[3] Department of Cell Biology and Histology, Faculty of Medicine and Nursing, University of Basque Country (UPV/EHU), 48940 Leioa, Spain
* Correspondence: mararabra@yahoo.co.uk; Tel.: +34 943 00 6108 (M.J.A.-B.)

A

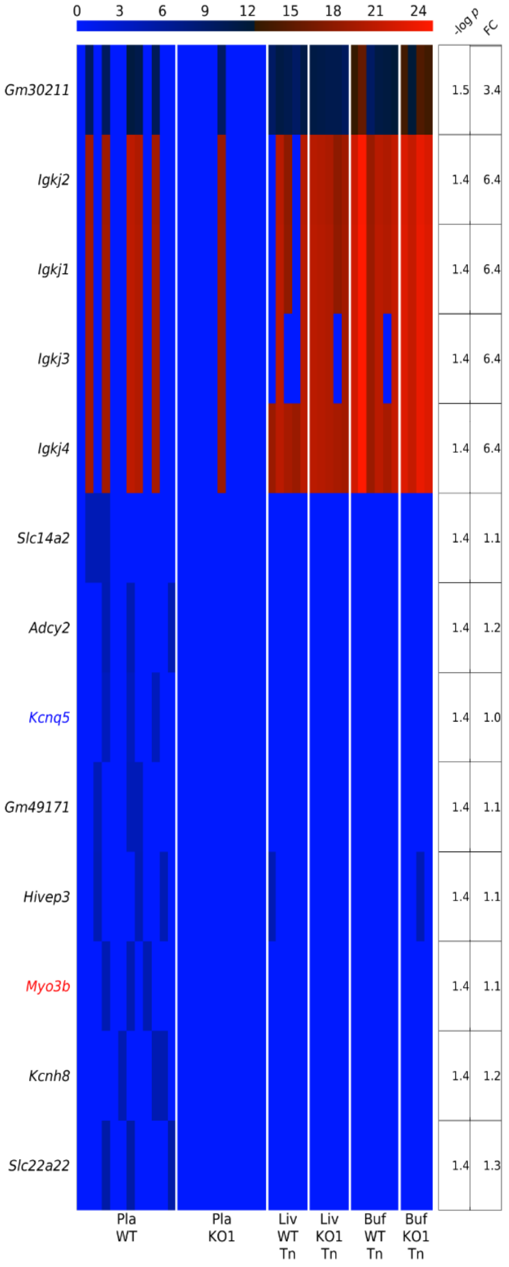

B

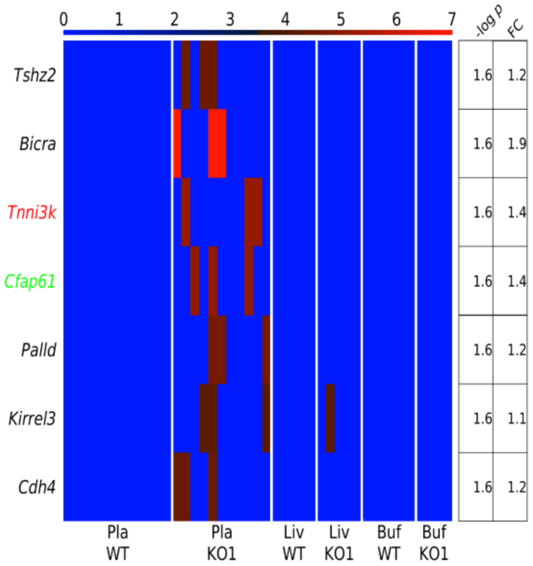

Figure S1. Heatmaps of the (**A**) 13 genes that led to up-DPpGCs in WT relative to Pla-KO1 and (**B**) 7 genes that led to up-DPpGCs in Pla-KO1 relative to WT, in decreasing order of significance. The PpGCs in the liver samples Liv-WT-Tn and Liv-KO1-Tn, and in the buffy coat samples But-WT-Tn and Buf-KO1-Tn, are given in the heatmaps as an additional comparison for the eccDNA production in the corresponding tissues based on libraries created with the tagmentation (Tn) based protocol. The color bars codify the value of PpGCs in $\log_2$ scale. Higher value corresponds to a redder color. The $-\log_{10}$ (*p*-value) and the absolute value of the $\log_2$ of the fold change (FC) of the DPpGCs are presented in a table to the right of the heat maps. The colored genes are associated to chromosomal fragile sites (CFS): red, green, blue, cyan, and magenta for CFSs induced by Aphidicolin, Folic acid, BrdU, 5-azacytidine, and DistamycinA, respectively.

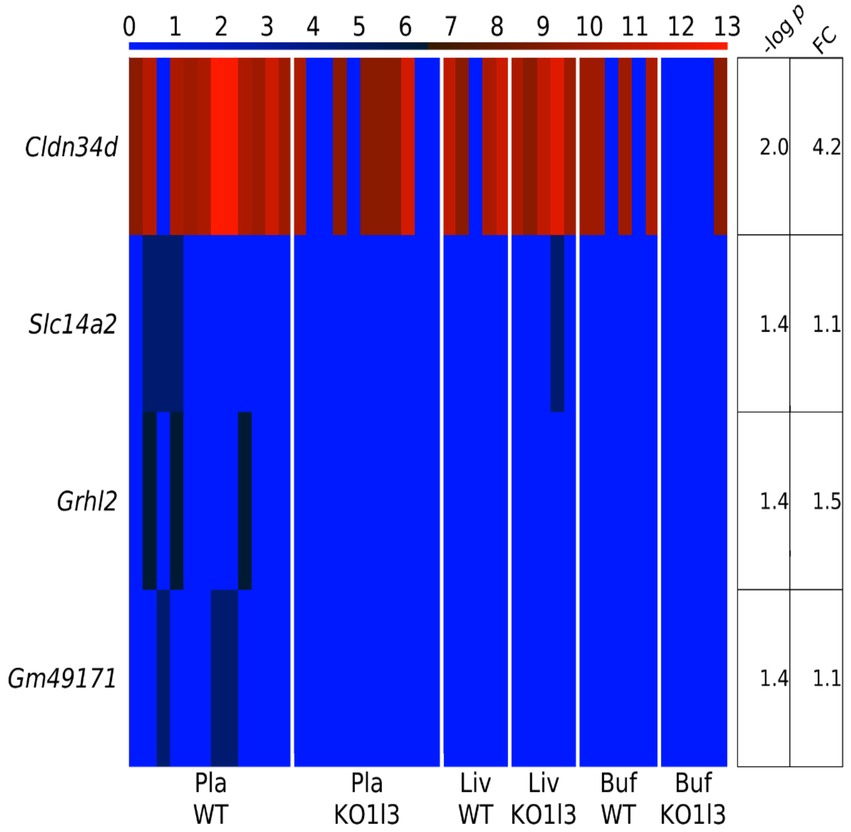

Figure S2. Heatmap of the four genes that led to up-DPpGCs in WT relative to Pla-KO1l3 in decreasing order of significance. The PpGCs in the liver samples Liv-WT-Tn and Liv-KO1l3-Tn, and in the buffy coat samples Buf-WT-Tn and Buf-KO1l3-Tn, are given in the heatmaps as an additional comparison for the eccDNA production in the corresponding tissues based on libraries created with the tagmentation (Tn) based protocol. The color bars codify the value of PpGCs in $\log_2$ scale. Higher value corresponds to a redder color. The $-\log_{10}$ (*p*-value) and the absolute value of the $\log_2$ of the fold change (FC) of the DPpGCs are presented in a table to the right of the heat map. The colored genes are associated to chromosomal fragile sites (CFS): red, green, blue, cyan, and magenta for CFSs induced by Aphidicolin, Folic acid, BrdU, 5-azacytidine, and DistamycinA, respectively.

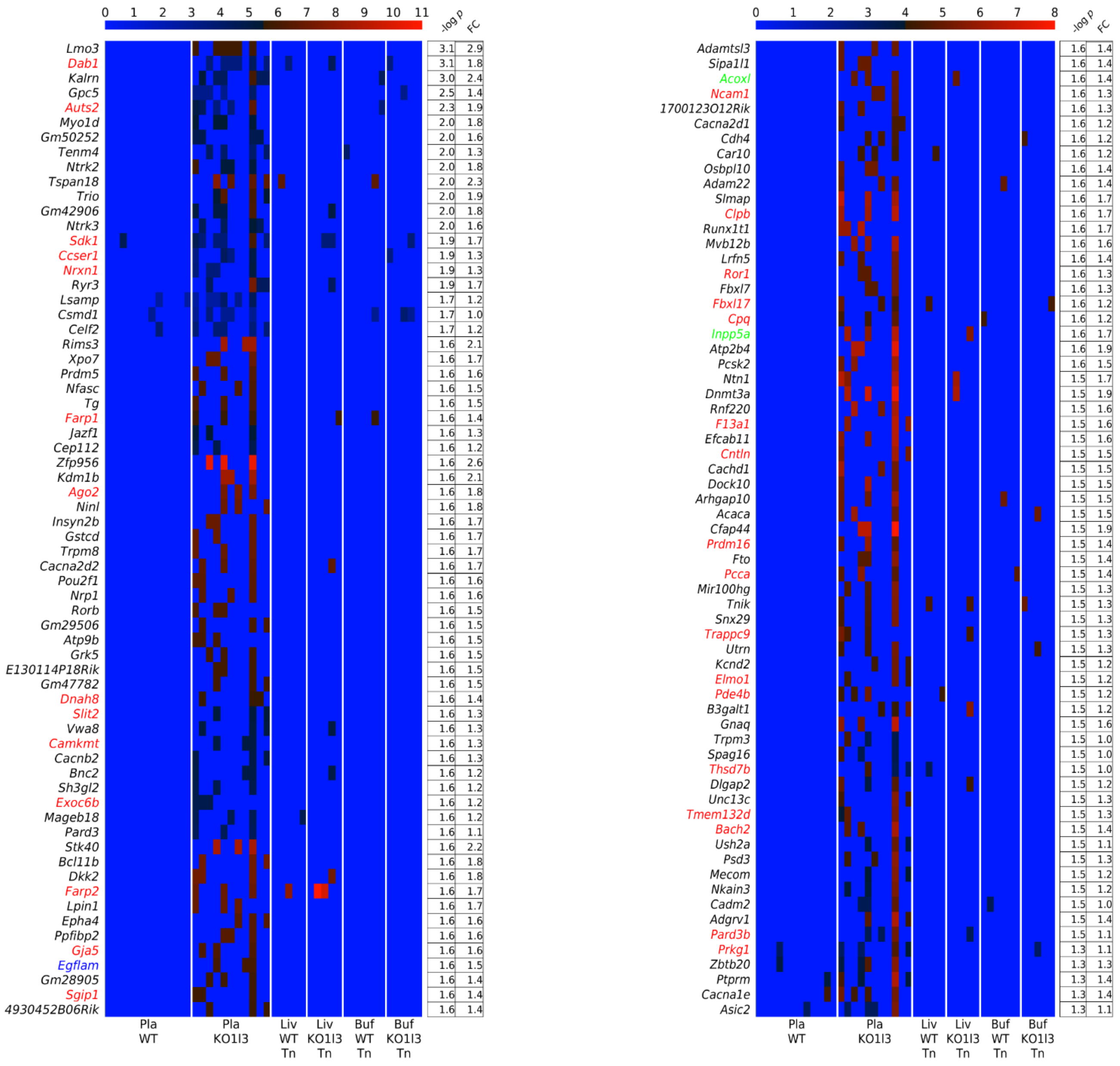

Figure S3. Heat maps of the 131 genes that led to up-DPpGCs in Pla-KO1l3 compared to WT in decreasing order of significance. The PpGCs in the liver samples Liv-WT-Tn and Liv-KO1l3-Tn, and in the buffy coat samples Buf-WT-Tn and Buf-KO1l3-Tn, are given in the heatmaps as an additional comparison for the eccDNA production in the corresponding tissues based on libraries created with the tagmentation (Tn) based protocol. The color bars codify the value of PpGCs in log_2 scale. Higher value corresponds to a redder color. The $-log_{10}$ (p-value) and the absolute value of the log_2 of the fold change (FC) of the DPpGCs are presented in a table to the right of the heat map. The colored genes are associated to chromosomal fragile sites (CFS): red, green, blue, cyan, and magenta for CFSs induced by Aphidicolin, Folic acid, BrdU, 5-azacytidine, and DistamycinA, respectively.

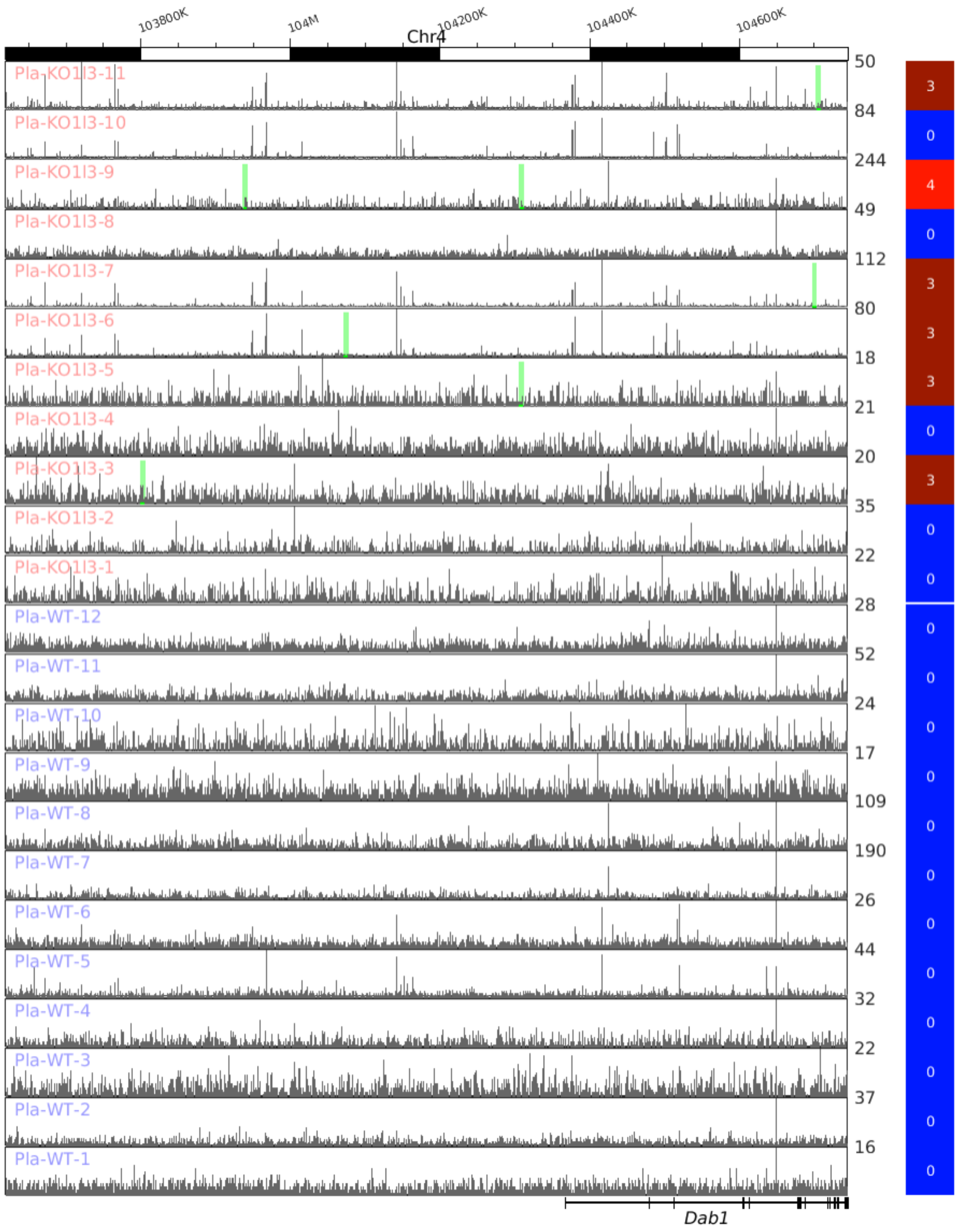

Figure S4. Track plots of the *loci* the 2nd top-ranked up-DPpGC in Pla-KO1l3 compared to WT, *Dab1*, and the corresponding gene coverage in all the samples in the two groups. Each horizontal line represents the length of a gene. The green bars represent the excision *loci* of the eccDNA. The color bars to the right codify the value of PpGCs in log_2 scale.

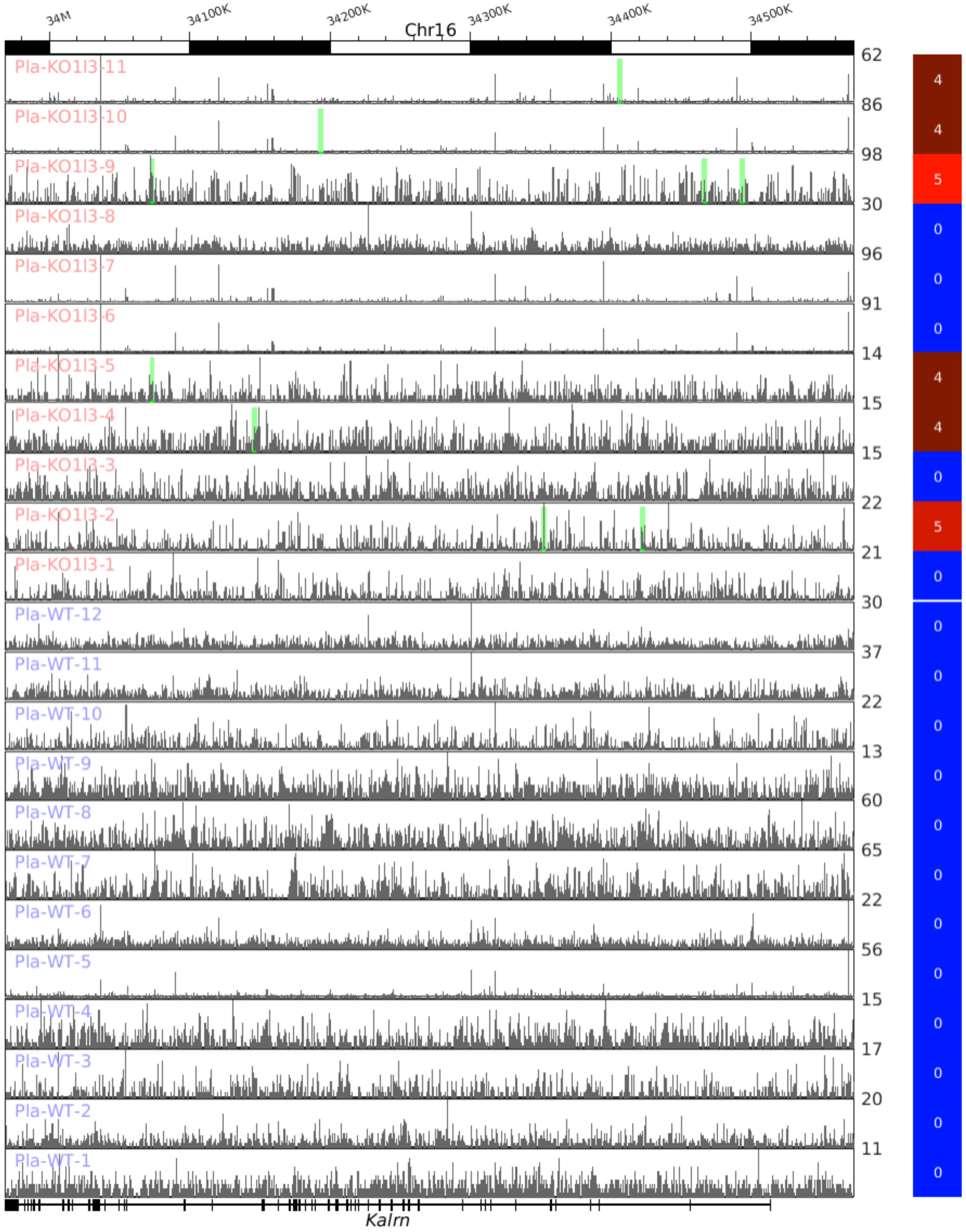

Figure 5. Track plots of the *loci* the 3rd top-ranked up-DPpGC in Pla-KO1l3 compared to WT, *Kalrn*, and the corresponding gene coverage in all the samples in the two groups. Each horizontal line represents the length of a gene. The green bars represent the excision *loci* of the eccDNA. The color bars to the right codify the value of PpGCs in $\log_2$ scale.

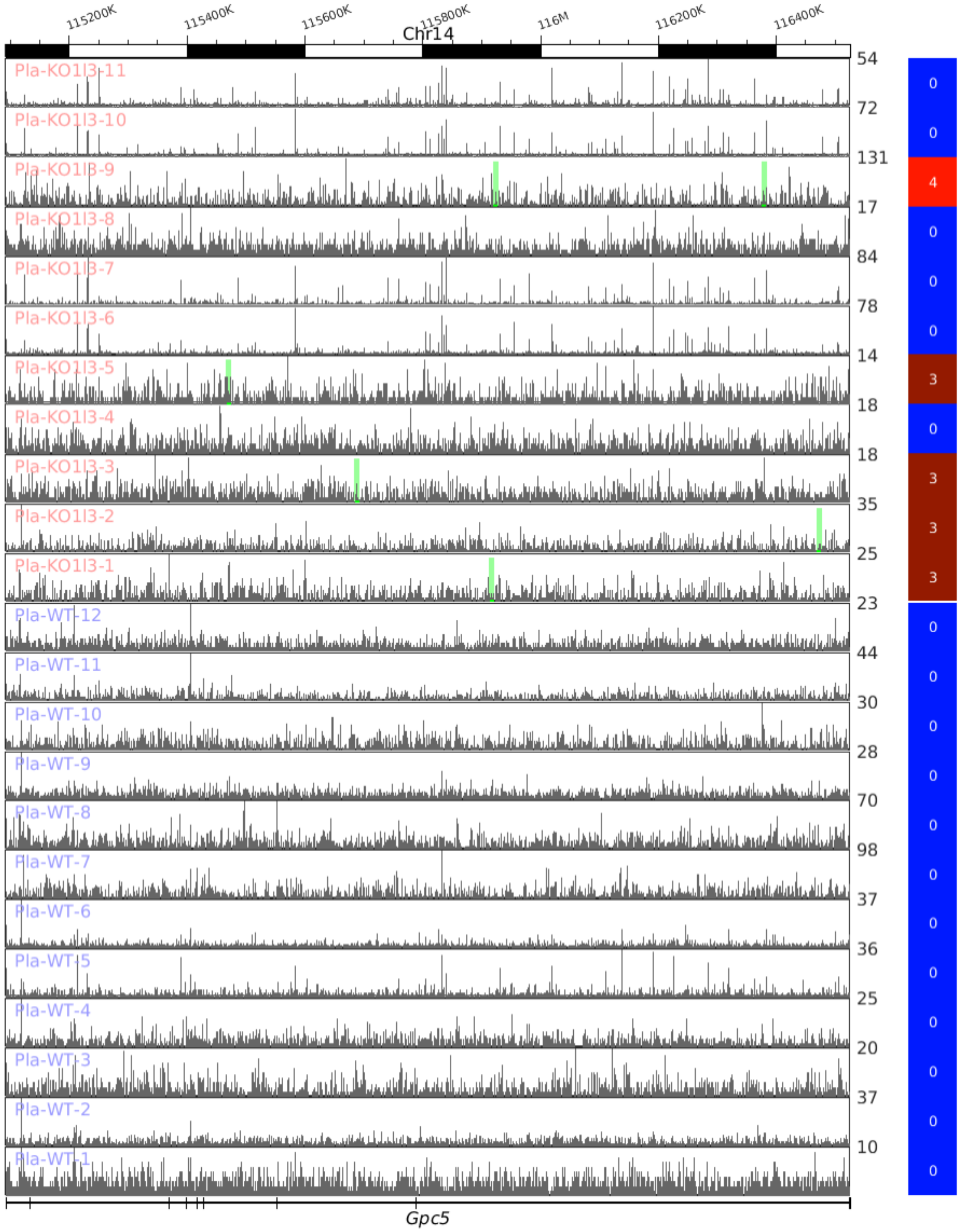

Figure S6. Track plots of the *loci* the 4th top-ranked up-DPpGC in Pla-KO1l3 compared to WT, *Gpc5*, and the corresponding gene coverage in all the samples in the two groups. Each horizontal line represents the length of a gene. The green bars represent the excision *loci* of the eccDNA. The color bars to the right codify the value of PpGCs in $\log_2$ scale.

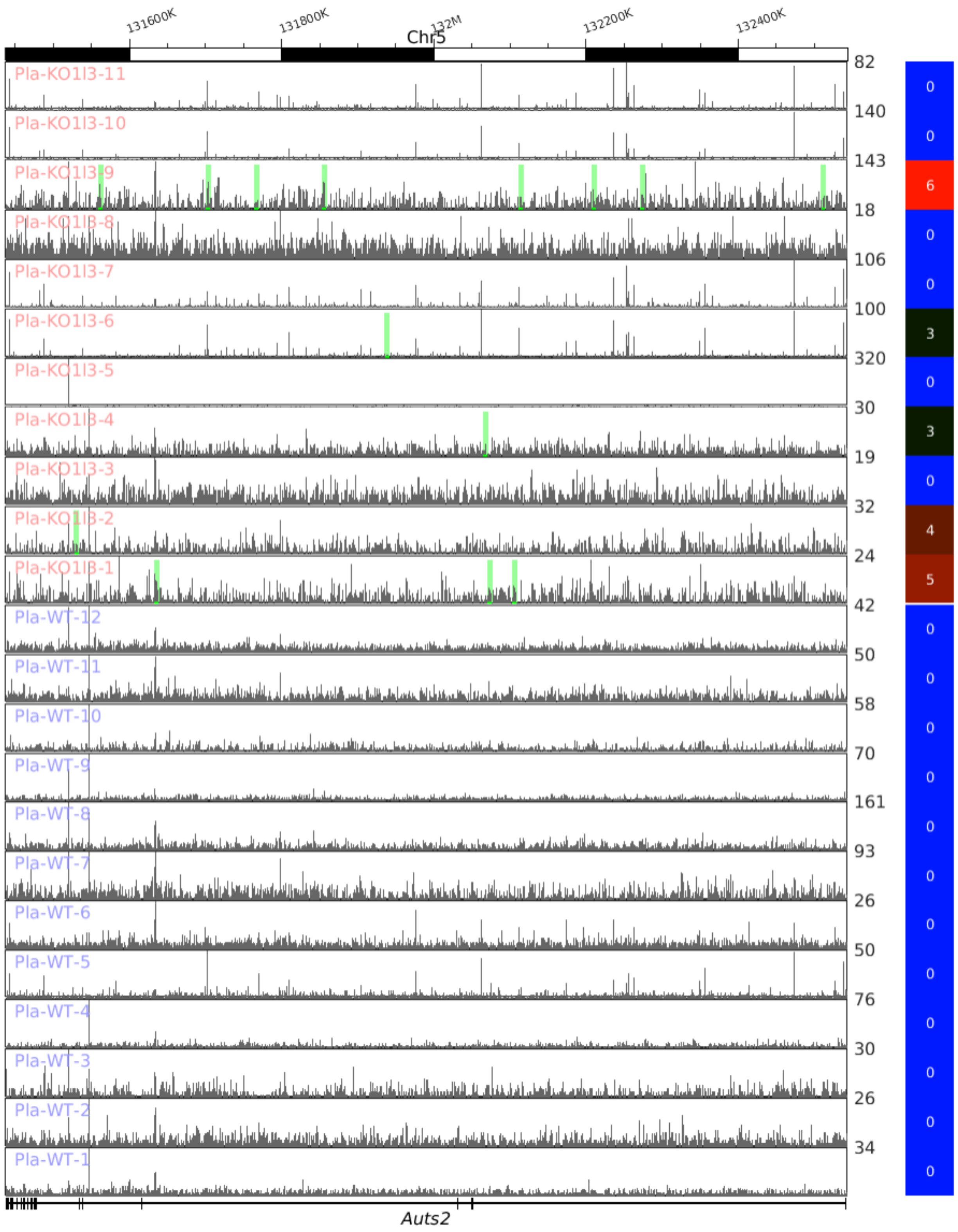

Figure S7. Track plots of the *loci* the 5th top-ranked up-DPpGC in Pla-KO1l3 compared to WT, *Auts2*, and the corresponding gene coverage in all the samples in the two groups. Each horizontal line represents the length of a gene. The green bars represent the excision *loci* of the eccDNA. The color bars to the right codify the value of PpGCs in $\log_2$ scale.

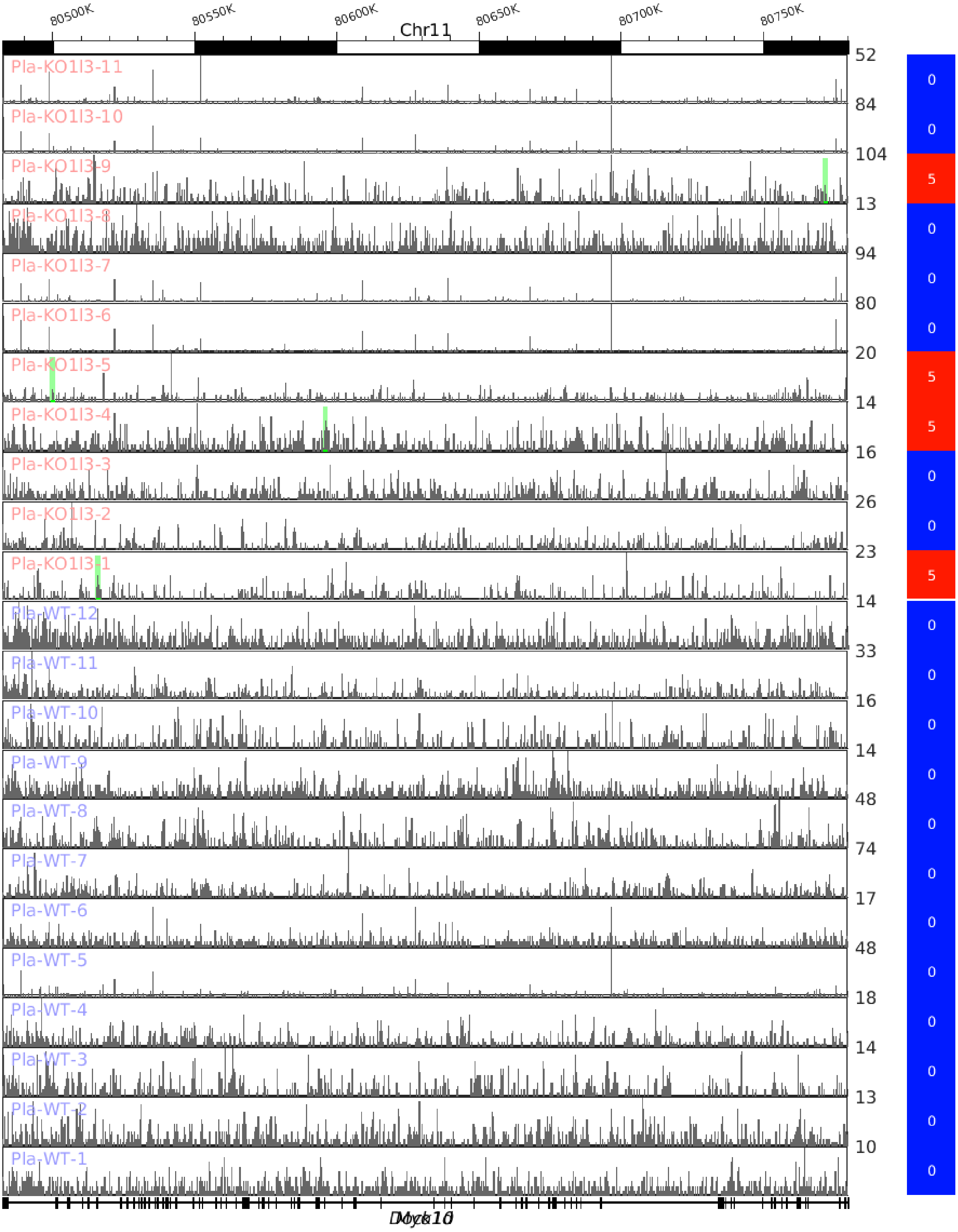

Figure S8. Track plots of the *loci* the 6th top-ranked up-DPpGC in Pla-KO1l3 compared to WT, *Myo1d*, and the corresponding gene coverage in all the samples in the two groups. Each horizontal line represents the length of a gene. The green bars represent the excision *loci* of the eccDNA. The color bars to the right codify the value of PpGCs in log_2 scale.

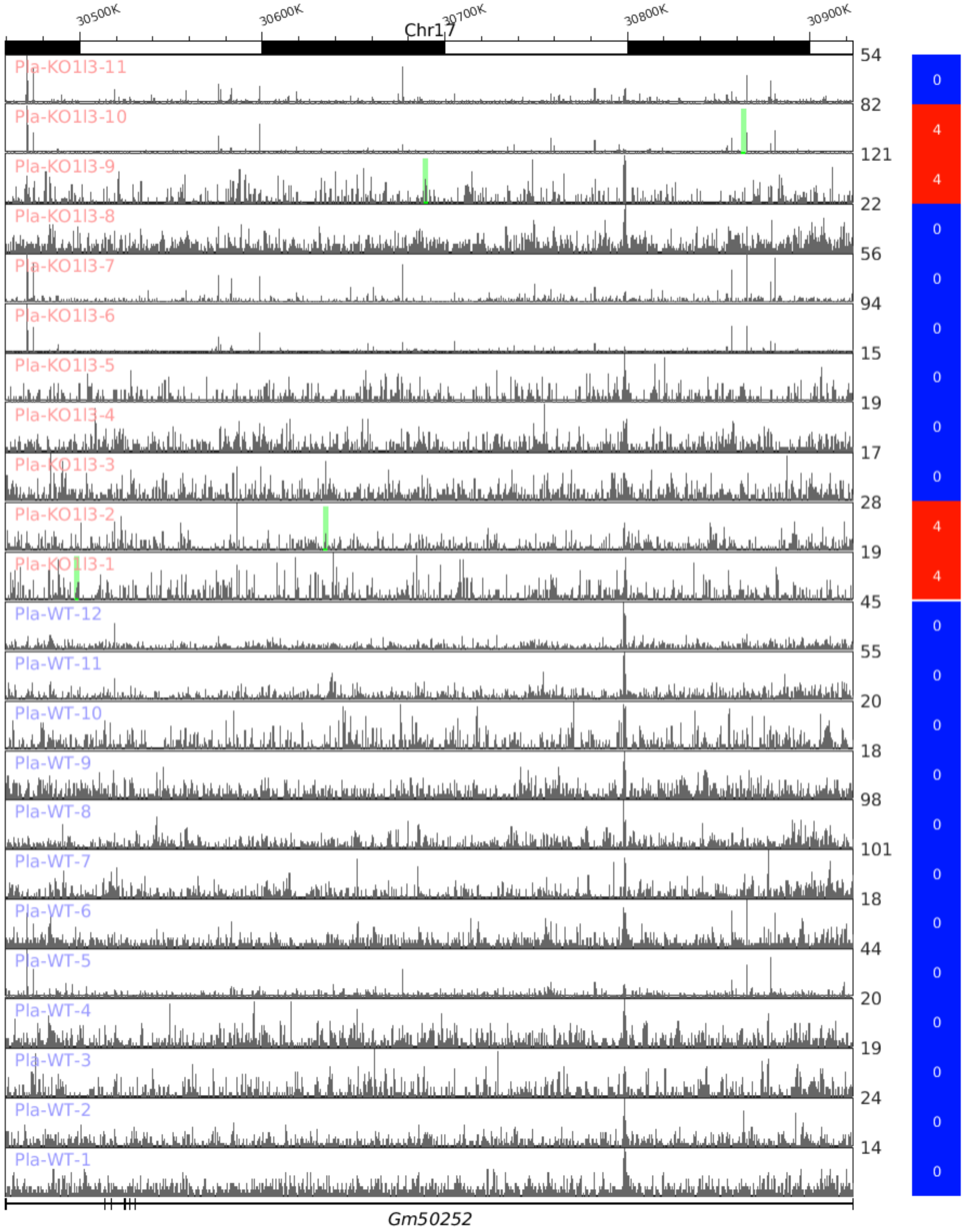

Figure S9. Track plots of the *loci* the 7th top-ranked up-DPpGC in Pla-KO1l3 compared to WT, *Gm50252*, and the corresponding gene coverage in all the samples in the two groups. Each horizontal line represents the length of a gene. The green bars represent the excision *loci* of the eccDNA. The color bars to the right codify the value of PpGCs in $\log_2$ scale.

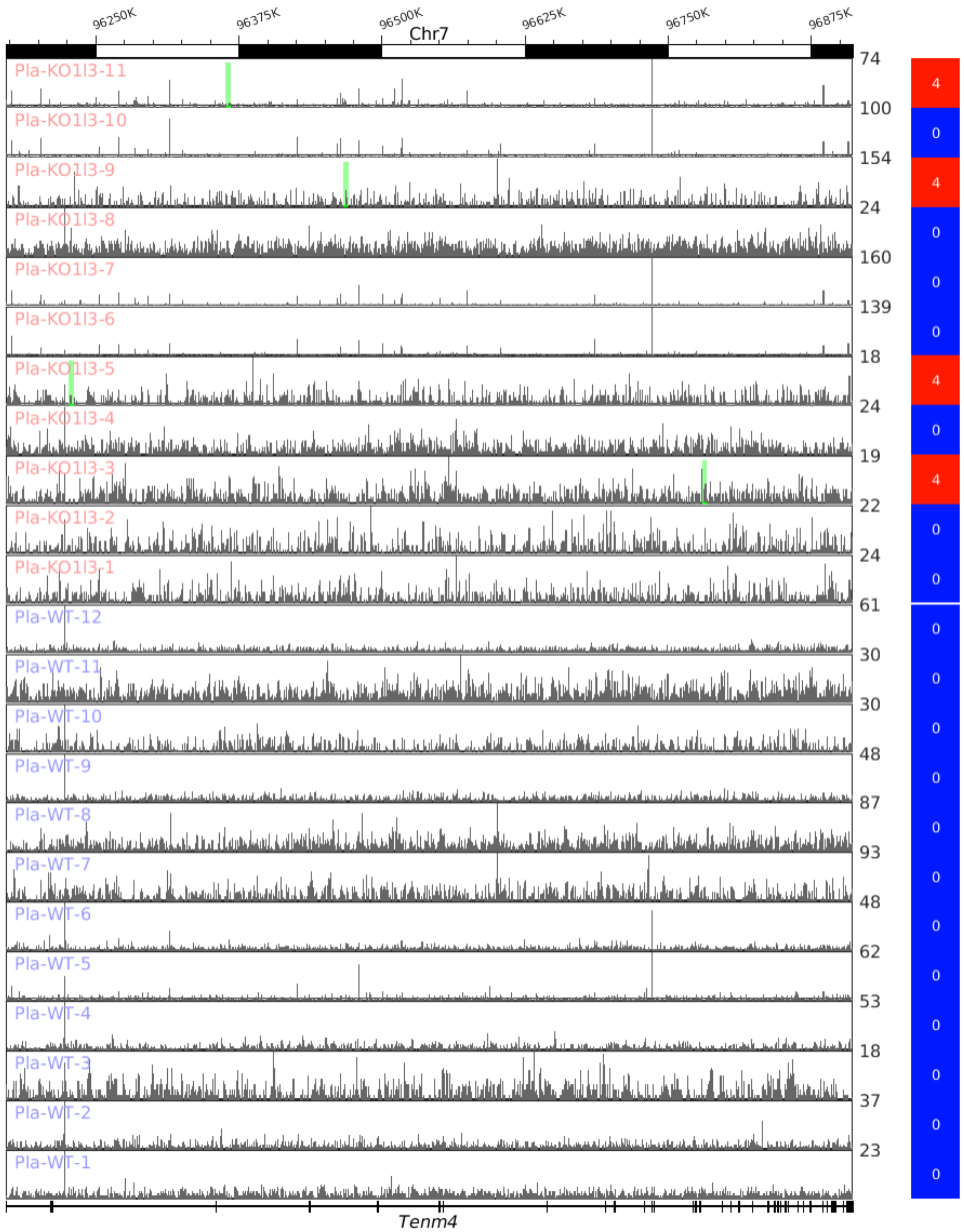

Figure S10. Track plots of the *loci* the 8th top-ranked up-DPpGC in Pla-KO1l3 compared to WT, *Tenm4*, and the corresponding gene coverage in all the samples in the two groups. Each horizontal line represents the length of a gene. The green bars represent the excision *loci* of the eccDNA. The color bars to the right codify the value of PpGCs in log_2 scale.

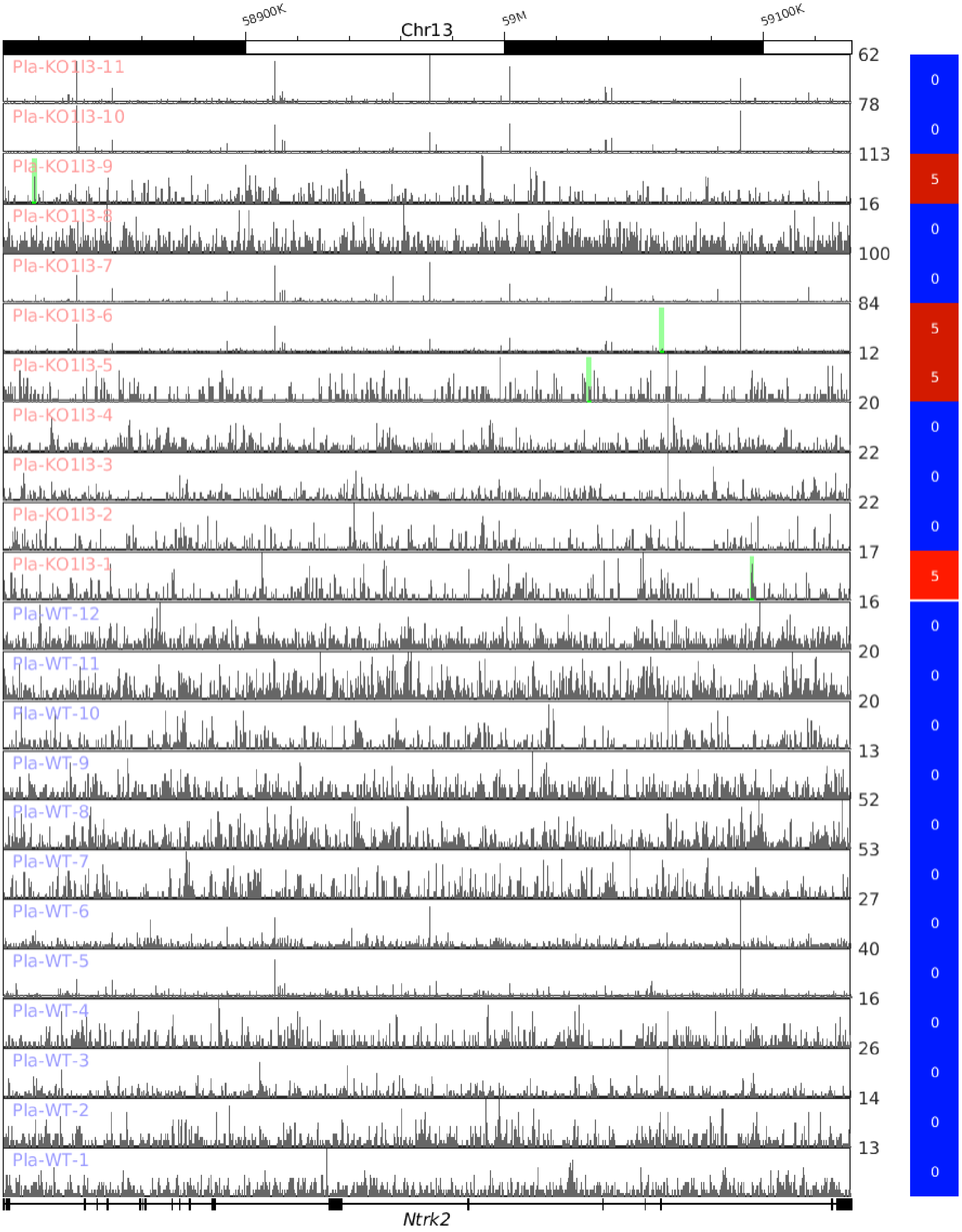

Figure S11. Track plots of the *loci* the 9th top-ranked up-DPpGC in Pla-KO1l3 compared to WT, *Ntrk2*, and the corresponding gene coverage in all the samples in the two groups. Each horizontal line represents the length of a gene. The green bars represent the excision *loci* of the eccDNA. The color bars to the right codify the value of PpGCs in $\log_2$ scale.

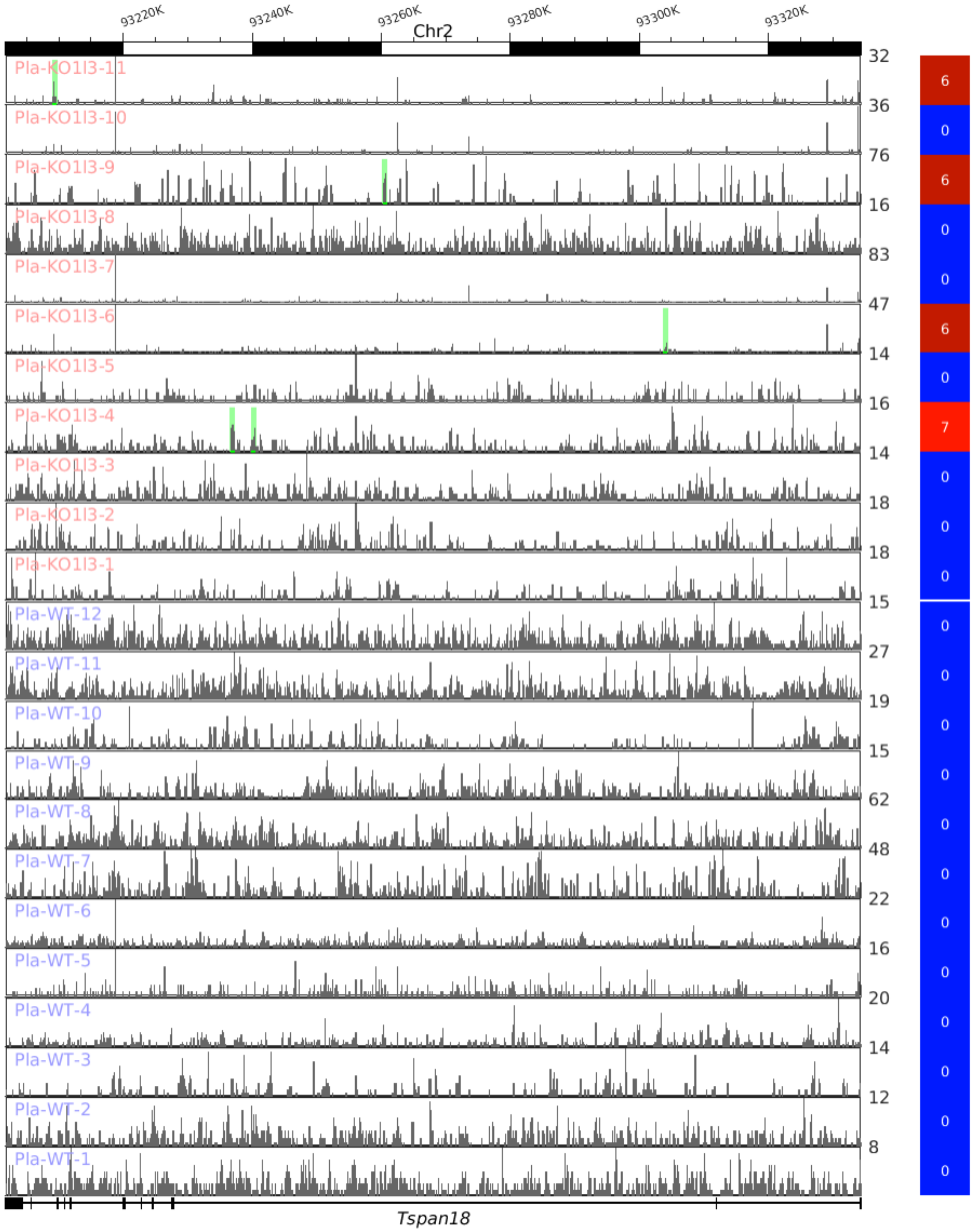

Figure S12. Track plots of the *loci* the 10th top-ranked up-DPpGC in Pla-KO1l3 compared to WT, *Tspan2*, and the corresponding gene coverage in all the samples in the two groups. Each horizontal line represents the length of a gene. The green bars represent the excision *loci* of the eccDNA. The color bars to the right codify the value of PpGCs in log_2 scale.

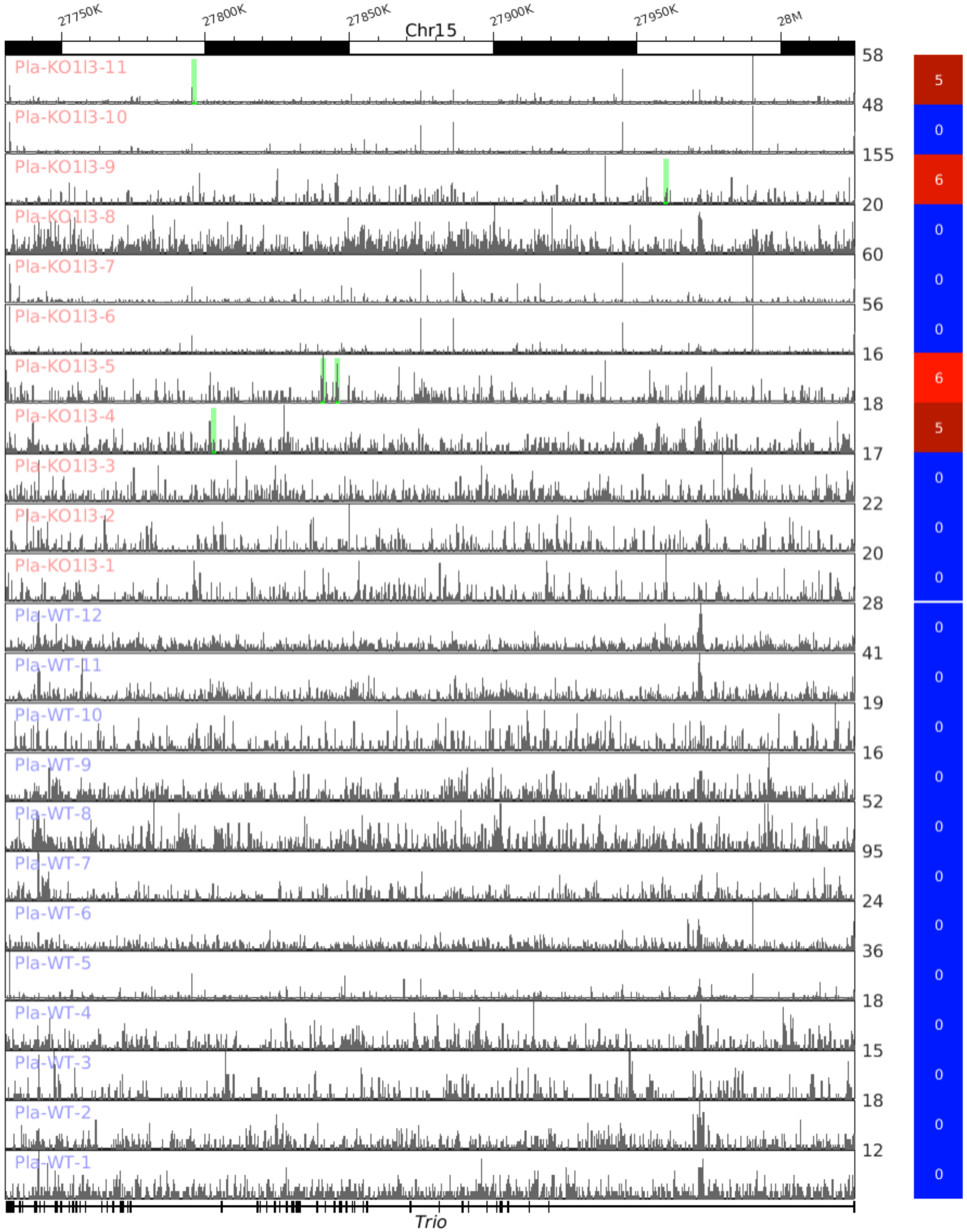

Figure S13. Track plots of the *loci* the 11th top-ranked up-DPpGC in Pla-KO1l3 compared to WT, *Trio*, and the corresponding gene coverage in all the samples in the two groups. Each horizontal line represents the length of a gene. The green bars represent the excision *loci* of the eccDNA. The color bars to the right codify the value of PpGCs in log_2 scale.

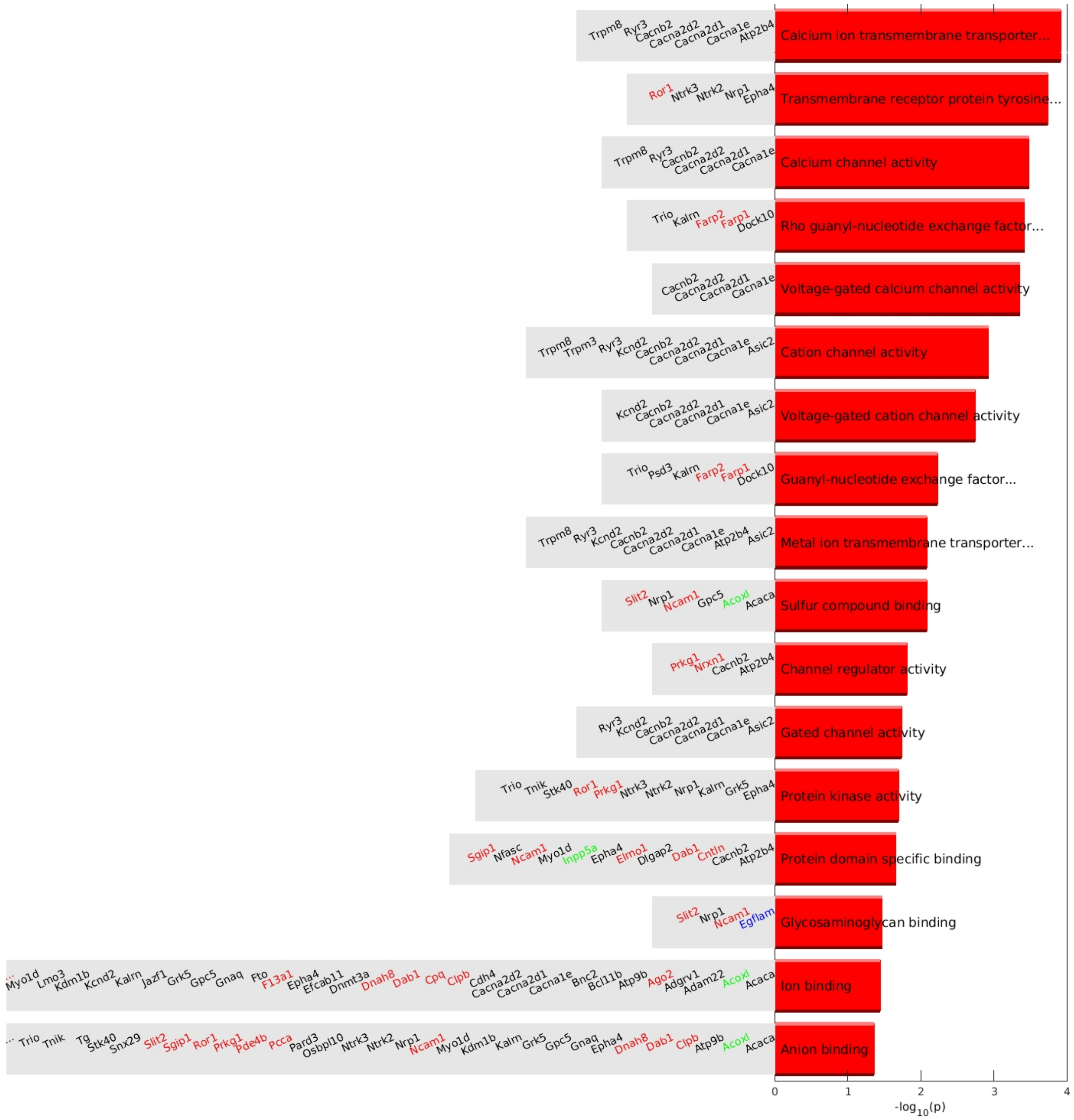

Figure S14. GO **Molecular function** terms enrichment analysis of up-DPpGCs in Pla-KO1l3. (Right) Bar plots of the –log10(*p*-value) of the significantly enriched terms. (Left) List of genes in the significant GO enrichment terms. The colored genes are associated to chromosomal fragile sites (CFS): red, green, blue, cyan, and magenta for genes associated to CFSs induced by Aphidicolin, Folic acid, BrdU, 5-azacytidine, and DistamycinA, respectively.

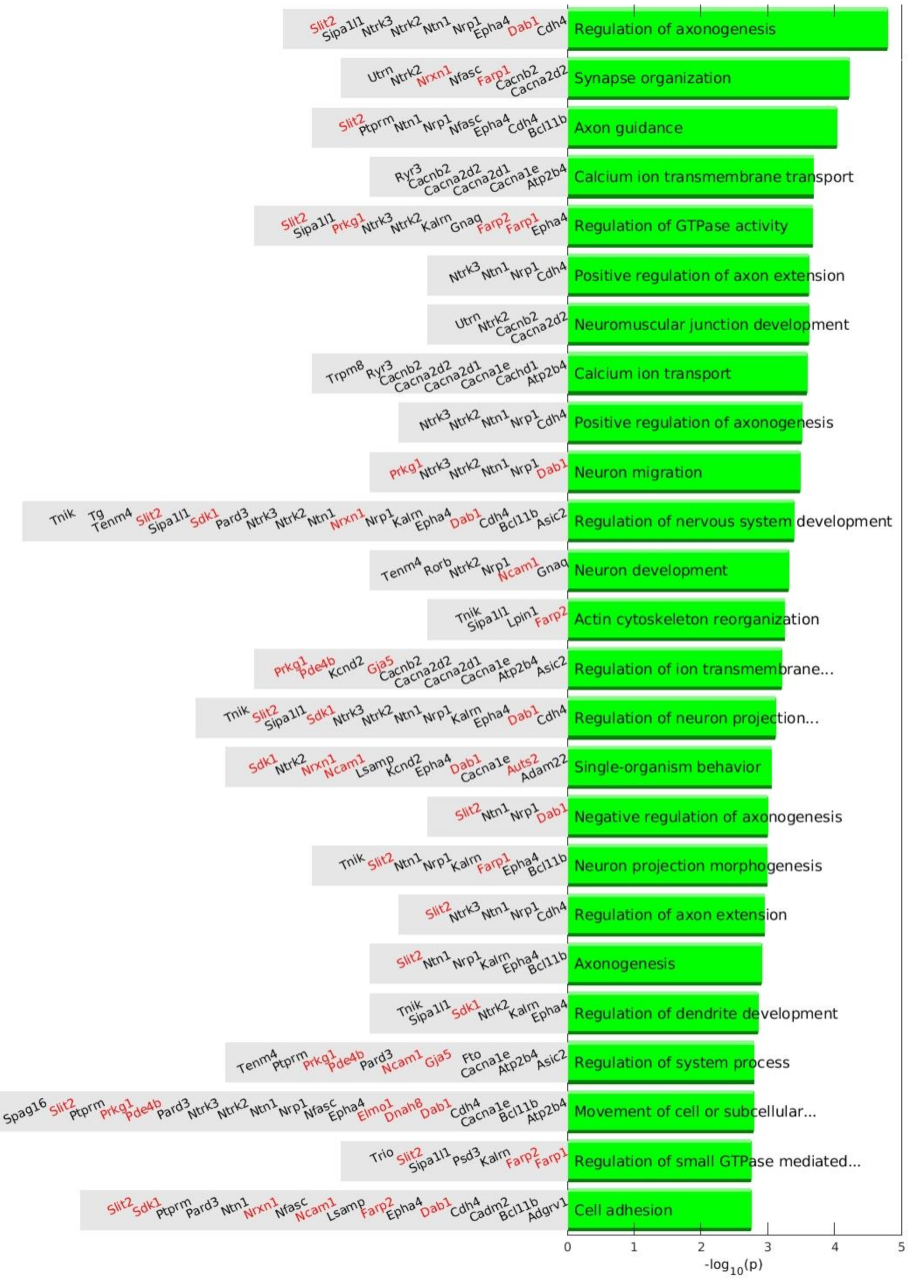

Figure S15. GO **Biological process** terms enrichment analysis of up-DPpGCs in Pla-KO1l3. (Right) Bar plots of the –log10(*p*-value) of the significantly enriched terms. (Left) List of genes in the significant GO enrichment terms. The colored genes are associated to chromosomal fragile sites (CFS): red, green, blue, cyan, and magenta for genes associated to CFSs induced by Aphidicolin, Folic acid, BrdU, 5-azacytidine, and DistamycinA, respectively.

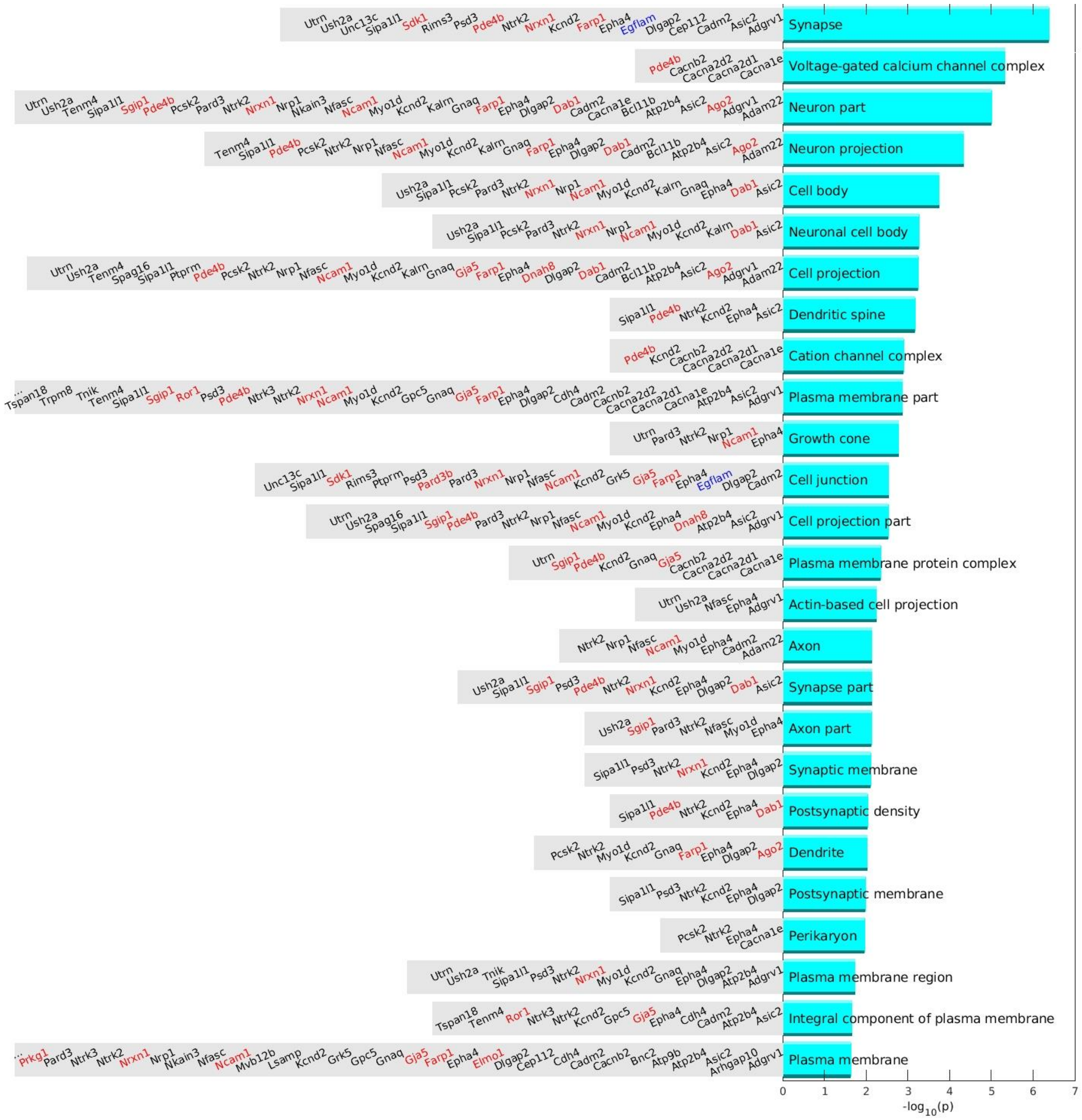

Figure S16. GO **Cellular component** terms enrichment analysis of up-DPpGCs in Pla-KO1l3. (Right) Bar plots of the –log10(*p*-value) of the significantly enriched terms. (Left) List of genes in the significant GO enrichment terms. The colored genes are associated to chromosomal fragile sites (CFS): red, green, blue, cyan, and magenta for genes associated to CFSs induced by Aphidicolin, Folic acid, BrdU, 5-azacytidine, and DistamycinA, respectively. The '…' mark the cases with more than 30 genes.
